# Altitude and case fatality rate from COVID-19 during the pandemic in Peru, 2020-2022

**DOI:** 10.17843/rpmesp.2026.431.14979

**Published:** 2026-03-17

**Authors:** Rodrigo Robles-Mariños, Diego Fano-Sizgorich, Germán F. Alvarado, Gustavo F. Gonzales

**Affiliations:** 1 School of Public Health and Administration, Universidad Peruana Cayetano Heredia, Lima, Peru.; 2 Endocrinology and Reproduction Laboratory, School of Science and Engineering, Department of Biological and Physiological Sciences, Universidad Peruana Cayetano Heredia, Lima, Peru.; 3 School of Health Sciences, Universidad San Ignacio de Loyola, Lima, Peru.

**Keywords:** COVID-19, Case Fatality Rate, Mortality, Altitude, Peru

## Abstract

**Objectives.:**

To evaluate the functional form of the association between the district altitude of residence and the case fatality rate of COVID-19 cases in Peru during the 2020-2022 period.

**Materials and methods.:**

A retrospective cohort study was conducted using secondary data from the Ministry of Health of Peru on COVID-19 cases and deaths. District altitude of residence was categorized into four groups (0-500; 501-2000; 2001-3500; and ≥350 masl). The outcome was death from COVID-19 according to the official death registry. Poisson regression was used to estimate adjusted relative risks for individual and contextual variables, using robust variance clustered by district. The analyses were repeated according to the five epidemic waves that occurred between March 2020 and December 2022.

**Results.:**

A non-linear association was observed between the altitude of residence and death from COVID-19, consistent with a "U-shaped" pattern, characterized by a lower risk of death in cases residing between 2001 and 3500 masl compared to those residing between 0 and 500 masl. This pattern remained consistent in the analyses stratified by epidemic wave.

**Conclusions.:**

In Peru, during the 2020-2022 period, the data do not support a monotonic decrease in the risk of death from COVID-19 with increasing altitude of residence. On the contrary, the results suggest a non-linear relationship, with lower lethality at intermediate altitudes (2001-3500 masl).

## INTRODUCTION

Peru reported its first cases of coronavirus disease 2019 (COVID-19) in March 2020, and in the following weeks, cases and deaths rapidly increased in many regions. Initially, the criterion for defining a COVID-19 death was based on the existence of a positive test prior to death ^1^. However, many deaths attributable to COVID-19 were not being recorded as such. To correct this underestimation, the Ministry of Health of Peru (MINSA) established seven more sensitive clinical-epidemiological criteria for recording COVID-19 deaths, which nearly tripled the number of deaths counted ^2^.

Mortality and case fatality due to COVID-19 vary according to individual characteristics. Older adults and males present a higher risk of death ^3^^,^^4^, as do people with comorbidities such as cancer, coronary disease, hypertension, obesity, diabetes, and respiratory and renal diseases ^5^^,^^6^. However, mortality also depends on contextual factors, such as the biosafety measures implemented and the capacity of healthcare systems ^7^^,^^8^. The introduction of vaccines constituted a key strategy to reduce mortality and case fatality ^9^. Simultaneous consideration of individual and contextual factors is essential to understand the determinants of COVID-19 case fatality, as focusing solely on one of these levels could lead to partial or biased conclusions.

Altitude has been identified as a factor potentially related to COVID-19 mortality, although the evidence is contradictory. Initial studies in Bolivia and Ecuador suggested a protective effect of altitude on both infection and death ^10^, and similar findings were reported in other international analyses ^11^^,^^12^. However, subsequent investigations found no association ^(^^13^^-^^16^^)^ or even reported a higher risk of death at high altitudes ^17^. Adjustments for comorbidities and sociodemographic variables indicated a decrease in case fatality with altitude in some studies ^18^. These discrepancies reflect the complexity of the phenomenon and the need for rigorous studies that consider multiple factors simultaneously ^19^^,^^20^.

In Peru, a country with great altitudinal variation, it is fundamental to evaluate the relationship between altitude and COVID-19 case fatality with a more detailed approach. Previous studies have used altitude at the provincial level or exclusively ecological designs ^16^^,^^20^. This study attempts to overcome those limitations by integrating an approach that combines individual factors (sex, age, vaccination, hospitalization) and contextual factors (district altitude, population density, human development index), which constitutes a more complete and robust methodological approach.

 The primary objective of this study was to evaluate the functional form of the association between district altitude of residence and COVID-19 case fatality in Peru during the 2020-2022 period. As a secondary objective, the study aimed to analyze this association according to the five epidemic waves that occurred in the country, considering the variation in health response capacity and the impact of vaccination strategies, both factors influenced by the emergence of different variants of the virus throughout the pandemic.

**KEY MESSAGES**

Motivation for the study. The relationship between COVID-19 death and altitude is controversial. In Peru, a country with great altitudinal variation, exploring this relationship is key to developing prevention and control strategies for this type of disease.

Main findings. A possible "U-shaped" association was found, where cases residing between 2001-3500 meters above sea level (masl) had a lower risk of death from COVID-19.

Public health implications. These findings highlight the need for a comprehensive approach that considers geographical factors along with effective preventive measures to address epidemics of this nature.

## MATERIALS AND METHODS

### Study design

A retrospective cohort study was conducted using open-access databases. The evaluation period was from March 2020 to December 2022.

### Study population

The study population consisted of COVID-19 cases registered in the MINSA database. Each case was considered individually as the unit of analysis. COVID-19 deaths without positive tests were also included in the study. A period of ±90 days was taken into account to consider a new episode of COVID-19. For deaths not previously reported as cases, the date of death was taken as the case date. Incomplete or inconsistent records that did not allow linking death and altitude data were excluded.

### Variables

COVID-19 death was the main dependent variable, based on the record in the MINSA database, which registers deaths according to any of the following seven criteria (2):

virological: presence of a positive virological test in the previous 60 days;

serological: a positive IgM or IgM/IgG serological test in the previous 60 days;

radiological: death of a probable case with radiological, tomographic, or nuclear magnetic resonance evidence compatible with COVID-19 pneumonia;

epidemiological link: death of a probable case presenting an epidemiological link with a confirmed case of COVID-19;

epidemiological investigation: death of a suspicious case verified by an epidemiological investigation;

clinical: death of a suspicious case presenting a clinical presentation compatible with COVID-19; and

SINADEF (National Computer System for Deaths): evidence from the death certificate in which the diagnosis of COVID-19 is presented as the cause of death.

For the purposes of the study, the variable only considered whether the case was present in said database, regardless of the criterion used for its inclusion.

The main exposure variable was the district altitude of residence and was categorized according to the physiological variations reported in the literature, opting for the generation of four categories: 0-500 masl (low altitude), 501-2000 masl (intermediate altitude), 2001-3500 masl (moderately high altitude), and ≥3501 masl (very high altitude). This classification was based on the first altitudinal floor, which was related to sea level (0-500 masl) ^21^; then, 2000 (masl) was established as the second cutoff point, used to establish a moderate/high altitude related to physiological changes ^22^^,^^23^ in possible relation to the protective effect of altitude. Finally, the cutoff point was established at 3500 masl, as altitudes above this are considered very high, related to maladaptation and pathological changes even in apparently healthy individuals ^24^.

Although altitude was categorized into the four aforementioned ranges based on physiological variations described in the literature for the main analysis, we recognize that this categorization implies a simplification of a continuous exposure and does not exactly correspond to the bioclimatic altitudinal floors of Peru (for example, according to the Pulgar Vidal classification) ^25^. This methodological decision was considered appropriate for the main analysis because it aligns with the reported inflection points in the physiological response to hypoxia. However, the possibility of non-differential misclassification of the exposure is recognized. Therefore, as part of a sensitivity analysis, an alternative recategorization of altitude more aligned with the altitudinal floors proposed by Pulgar Vidal was performed to evaluate the robustness of the observed pattern under an ecological scheme traditionally applied to the Peruvian context. Thus, the following categories were generated: 0-500 masl, 501-2300 masl, 2301-3500 masl, 3501-4000 masl, and ≥4001 masl.

Regarding individual-level covariates, sex was taken into account, reported as male or female. Age was categorized into groups based on the risk of COVID-19 death according to reports from the Centers for Disease Control and Prevention (CDC), establishing the categories: 0-49 years, 50-74 years, and ≥75 years. COVID-19 vaccination was evaluated considering the record of immunization doses at the case date, generating the categories "unvaccinated," "1 dose" (incomplete schedule), "2 doses" (complete schedule), and "3 or more doses" (complete schedule + booster doses). In addition, COVID-19 hospitalization data was used as a proxy for infection severity, and a time period of ±90 days from the report date as a case was taken to consider hospitalization as part of the reported episode.

Regarding residential district-level covariates, the human development index (HDI), population density, and availability of health resources were obtained. Concerning the HDI, the data was attributed considering the report of the Peruvian Institute of Economics for each district. Then, according to the categories proposed by the United Nations, the classification was generated: low HDI (<55.0%), medium HDI (55.0-69.9%), high HDI (70.0-79.9%), and very high HDI (>80.0%). Population density was calculated by dividing the estimated total population per district according to the National Institute of Statistics and Informatics (INEI) for 2020 by the district area (in km^2^). It was then categorized according to the categories proposed in a study evaluating the efficiency of the health system in prevention and treatment against COVID-19 ^26^: low density (≤100 inhabitants/km^2^), medium density (>100 and ≤500 inhabitants/km^2^), and high density (>500 inhabitants/km^2^). The availability of health resources consisted of the availability of hospitalization beds, intensive care unit (ICU) beds, intermediate care unit (IMCU) beds, and emergency stretchers. These data were obtained from the records of the National Superintendence of Health (SUSALUD) of Peru, which provided a record of the availability of these resources for each health facility, although a complete record for the entire study was not available.

Finally, the epidemic wave variable was generated considering as the cutoff point the beginning of each of the 5 epidemic waves that occurred in Peru until December 2022, according to the dates declared by the National Center for Epidemiology, Prevention and Control of Diseases (CDC-Peru) and MINSA, published in COVID-19 situational rooms and in the official newspaper of Peru, El Peruano. The dates considered as the start for each epidemic wave were: 03/06/2020 for the 1st; 11/30/2020 for the 2nd; 01/04/2022 for the 3rd; 06/27/2022 for the 4th; and 12/02/2022 for the 5th epidemic wave. We recognize that the spread of SARS-CoV-2 in Peruvian territory was geographically heterogeneous and asynchronous, and that these dates do not necessarily reflect the local epidemic onset or peak in each district. However, there are no standardized definitions of epidemic waves at the regional or district level for the entire study period, so this approach was chosen for descriptive and temporal stratification purposes.

### Data collection

The databases (xls, xlsx, or csv) were downloaded and cleaned in STATA 18.0 for Windows. Then, the COVID-19 cases and deaths databases were merged using the unique identifier and the dates of infection and death. In this way, deaths not previously considered as cases were incorporated as new records, obtaining a database with the following data for each record: unique identifier, sex, age group, case date, and district of residence geographic location code (UBIGEO). An additional variable, “deceased case,” was included and coded as “yes” when a death was recorded during the infection period (±90 days). Based on the UBIGEO of residence, district altitude, HDI, and population density were assigned, adding these data as additional columns to the aforementioned database and for each record individually. Subsequently, vaccination status and hospitalization were added according to the unique identifier and case date as two additional columns, such that if the case had at least one immunization at the case date, this was reported as "yes vaccinated," including the doses received up to that point; and if a COVID-19 hospitalization was reported in the ±90-day period from the case date, it was reported as "yes hospitalized." Finally, according to the case date, data corresponding to the availability of health resources by district of residence were added, with the data for each record added as an additional column, and the epidemic wave category was established, adding this data as a final additional column.

The data from the databases used spanned the period from March 2020 to December 2022 and were obtained from the following sources with the following considerations:

COVID-19 cases: the MINSA database (dynamic database) was used for cases reported in Peruvian territory, taking into account the ±90-day period to consider a report of the same person in this database as a new COVID-19 episode, unifying records within this same period (considering only the first report as the indicator of the date the person was considered a case).

https://www.datosabiertos.gob.pe/dataset/casos-positivos-por-covid-19-ministerio-de-salud-minsa

COVID-19 deaths: the MINSA database (dynamic database including deaths according to the 7 criteria previously exposed) was also used, which was superimposed on the COVID-19 cases database according to the unique identifier. In cases of deaths not previously reported as cases, these were incorporated as COVID-19 cases, including the date of death as the date the person was considered a case (this occurred, for example, for deaths by investigation and epidemiological link criteria, where there were no positive tests to determine the onset of the disease, but the death was determined to be a result of said disease).

https://www.datosabiertos.gob.pe/dataset/fallecidos-por-covid-19-ministerio-de-salud-minsa

Altitude by district: the district altitude of residence was taken into account according to the reported district (obtained from the UBIGEO codes reported for each district) in the cases database first, complemented by the district of residence from the deaths database if the data was not obtained in the first database. The district of residence was reported according to the National Registry of Identification and Civil Status (RENIEC), which was linked to the district altitude reported in the MINSA database, which obtained information from INEI.

https://www.datosabiertos.gob.pe/dataset/codigos-equivalentes-de-ubigeo-del-peru

COVID-19 vaccination: the MINSA database (dynamic database) was used, which included the report of all COVID-19 immunizations individually and according to the unique identifier of each person, including the date of administration of each dose (which allowed knowing the vaccination status when compared with the case date). https://www.datosabiertos.gob.pe/dataset/vacunacion

COVID-19 hospitalizations: the MINSA database (dynamic database) was used, which included reports of hospitalizations in the national territory according to the unique identifier, including admission dates (which allowed knowing if the case was hospitalized during the COVID-19 episode, according to the case date). https://www.datosabiertos.gob.pe/dataset/hospitalizados-vacunados-y-fallecidos-por-covid-19

Population density: based on the district of origin, population density was estimated from the total population per district estimated by INEI for the year 2020, which was divided by district area (in km2) to obtain the final value included in the database. https://www.minsa.gob.pe/reunis/data/poblacion_estimada.asp District area data was obtained from the same database containing district altitudes mentioned above, with these data obtained from INEI reports. https://www.datosabiertos.gob.pe/dataset/codigos-equivalentes-de-ubigeo-del-peru

Human Development Index: this data was attributed considering the 2019 Peruvian Institute of Economics report for each district in the national territory. https://www.ipe.org.pe/portal/indice-de-desarrollo-humano-idh/

Available hospitalization beds, ICU, IMCU beds, and emergency stretchers: these data were obtained considering the record of SUSALUD of Peru (dynamic database), which provided a record of hospitalization, ICU, IMCU beds, and emergency stretchers available per each health facility, indicating the district to which each facility belonged. In this way, a record of health resources available to receive patients by district could be maintained. This record was conducted daily, but unfortunately, a complete record for the entire study period was not available (reporting was inconsistent across different health facilities throughout the COVID-19 pandemic, and there was no report for the periods during the first or fifth epidemic waves in Peru).

http://datos.susalud.gob.pe/dataset/data-hist%C3%B3rica-del-registro-de-camas-diarias-disponibles-y-ocupadas-del-formato-f5002-v2-2#{} 

The data come from the official records of MINSA, which depend on regional epidemiological surveillance systems. Given that the degree of notification may vary between jurisdictions, especially in remote or higher altitude districts, we recognize the possibility of differences in the completeness of the registry of cases and deaths. This potential variability in capture was considered in the interpretation of results and is discussed as a possible source of selection bias.

### Statistical analysis

First, a descriptive analysis was performed, reporting frequencies and percentages. A specific descriptive analysis was also conducted by epidemic waves and altitude categories.

Then, a bivariate analysis was performed to evaluate the relationship of COVID-19 death with altitude, as well as the relationship of COVID-19 death with sex, age group, vaccination, hospitalization, population density, and HDI, using the Chi-square test. This was performed globally and by epidemic waves.

Poisson regression with robust variance clustered by district was used, taking COVID-19 death as the outcome, estimating the relative risk (RR) with 95% confidence intervals (95%CI). The covariates to adjust the multivariable model were: sex, age group, vaccination, hospitalization, HDI, and population density. Global and epidemic wave analyses were performed. The global analysis was also adjusted for epidemic wave, and in the case of the multiple regression of the first epidemic wave, vaccination was not used because national vaccination had not yet begun. The assumption of intra-cluster dependence was taken into account, recognizing that observations within the same district could be correlated, but assuming independence between districts, and multicollinearity was evaluated using the variance inflation factor (VIF) through an auxiliary linear model with the same predictors as the main model.

Given that there was a large amount of missing data for the registry of available health resources, it was decided to exclude them from the main analysis. However, a sensitivity analysis was performed including the availability of health resources in the regressions (globally and by epidemic waves). Likewise, a sensitivity analysis was performed to evaluate only the deaths of cases with previous positive tests. Thus, regressions were repeated eliminating deaths that were not initially registered as cases, both globally and by epidemic waves. Finally, an additional sensitivity analysis was performed evaluating the relationship between altitude and COVID-19 death considering the alternative altitude classification more aligned with the altitudinal floors of Pulgar Vidal, repeating the regressions both globally and by epidemic waves.

A p-value <0.05 was considered statistically significant for all analyses performed. To carry out the described analysis plan, STATA 18.0 for Windows (Stata Corp., College Station, TX) was used.

### Ethical aspects

The study used open-access secondary databases. The identification variable came from an encrypted code, so there was no possibility of identifying the person included in the records. This protocol was registered in the Decentralized System of Information and Research Monitoring (SIDISI) - University Directorate of Research, Science and Technology (DUICT), under code 213552, and was evaluated by the UPCH Ethics Committee (CIE-UPCH) prior to execution, being approved under code CIEI-109-10-24. Likewise, registration was performed on the Health Research Projects platform (PRISA) under code EI00003486. During the development of the study, the ethical principles outlined in the Declaration of Helsinki were respected, and the recommendations provided by CIE-UPCH were strictly followed.

## RESULTS

A total of 4,331,784 COVID-19 cases were obtained in Peru between March 2020 and December 2022, but 211,015 records were excluded because they lacked a unique identifier (2.0%) and/or district of residence (2.9%), resulting in a total of 4,120,769 analyzable cases.

Of the analyzable total, 51.6% were women, 68.9% were under 50 years of age, and 53.0% had not been immunized when they had COVID-19. The largest proportion resided in a district with a medium HDI (45.2%) and high population density (66.5%). Likewise, the majority resided between 0 and 500 masl (69.9%). Finally, 215,423 deaths (5.2%) were recorded. This information, both globally and by epidemic waves, is shown in Table 1. Likewise, the characteristics described for each altitude category are presented in Supplementary Material 1.

In the bivariate analysis, a statistically significant association (p<0.001) was found between COVID-19 death and altitude, showing that people residing between 0 and 500 masl, as well as those residing from 3501 masl and above, reported the most deaths (5.7% and 5.6%, respectively). A statistically significant association (p<0.001) was also found with the variables of sex, age group, vaccination, hospitalization, HDI, and population density. These results are presented in Table 2. All presented associations were also evaluated by epidemic waves, which can be viewed in Table 3.

**Table 1:** Characteristics of COVID-19 patients overall and by epidemic wave, Peru 2020-2022.

Characteristics		Total	Epidemic wave				
			1st wave	2nd wave	3rd wave	4th wave	5tth wave
		n=4 120 769 (%)	n=952 811(%)	n= 1 280 781(%)	n=1 171 485 (%)	n=567 318(%)	n=148 374(%)
Sex							
	Female	2 124 796 (51.6)	458 153 (48.1)	633 048 (49.4)	627 615 (53.6)	321 576 (56.7)	84 404 (56.9)
	Male	1 995 973 (48.4)	494 658 (51.9)	647 733 (50.6)	543 870 (46.4)	245 742 (43.3)	63 970 (43.1)
Age group*							
	0 to 49 years	2 838 887 (68.9)	609 792 (64.0)	852 074 (66.5)	898 626 (76.7)	387 433 (68.3)	90 962 (61.3)
	50 to 74 years	1 078 263 (26.2)	283 661 (29.8)	356 519 (27.8)	235 082 (20.1)	154 889 (27.3)	48 112 (32.4)
	75 years and older	203 469 (4.9)	59 336 (6.2)	72 063 (5.6)	37 775 (3.2)	24 995 (4.4)	9 300 (6.3)
Vaccination							
	No	2 183 238 (53.0)	952 809 (∼100.0)	1 110 002 (86.7)	99 824 (8.5)	17 590 (3.1)	3 013 (2.0)
	1 dose	110 853 (2.7)	0 (0.0)	48 765 (3.8)	46 260 (4.0)	13 693 (2.4)	2 135 (1.4)
	2 doses	815 739 (19.8)	2 (∼0.0)	115 313 (9.0)	636 415 (54.3)	55 559 (9.8)	8 450 (5.7)
	3 or more doses	1 010 939 (24.5)	0 (0.0)	6 701 (0.5)	388 986 (33.2)	480 476 (84.7)	134 776 (90.8)
Hospitalization							
	No	4 038 276 (98.0)	922 674 (96.8)	1 235 012 (96.4)	1 166 472 (99.6)	566 047 (99.8)	148 071 (99.8)
	Yes	0000000000082 493 (2.0)	30 137 (3.2)	45 769 (3.6)	5 013 (0.4)	1 271 (0.2)	303 (0.2)
Human development index							
	Low	600 005 (14.6)	160 514 (16.8)	225 903 (17.6)	143 983 (12.3)	55 831 (9.8)	13 774 (9.3)
	Medium	1 861 734 (45.2)	471 486 (49.5)	571 442 (44.6)	524 852 (44.8)	226 670 (40.0)	67 284 (45.4)
	High	1 146 023 (27.8)	252 973 (26.6)	344 652 (26.9)	333 414 (28.5)	172 042 (30.3)	42 942 (28.9)
	Very high	513 007 (12.4)	67 838 (7.1)	138 784 (10.8)	169 236 (14.4)	112 775 (19.9)	24 374 (16.4)
Population density							
	Low	718 106 (17.4)	193 364 (20.3)	249 515 (19.5)	181 643 (15.5)	73 228 (12.9)	20 356 (13.7)
	Medium	661 123 (16.0)	165 586 (17.4)	213 710 (16.7)	184 626 (15.8)	74 592 (13.2)	22 609 (15.2)
	High	2 741 540 (66.5)	593 861 (62.3)	817 556 (63.8)	805 216 (68.7)	419 498 (73.9)	105 409 (71.0)
District altitude of residence							
	0 a 500 masl	2 878 797 (69.9)	693 839 (72.8)	859 704 (67.1)	818 372 (69.9)	401 296 (70.7)	105 586 (71.2)
	501 a 2000 masl	350 773 (8.5)	88 908 (9.3)	118 107 (9.2)	92 316 (7.9)	40 093 (7.1)	11 349 (7.6)
	2001 a 3500 masl	745 509 (18.1)	136 273 (14.3)	252 788 (19.7)	219 335 (18.7)	110 162 (19.4)	26 951 (18.2)
	3501 masl and more	145 690 (3.5)	33 791 (3.6)	50 182 (3.9)	41 462 (3.5)	15 767 (2.8)	4 488 (3.0)
COVID-19 death							
	No	3 905 346 (94.8)	859 572 (90.2)	1 171 565 (91.5)	1 161 665 (99.2)	564 630 (99.5)	147 914 (99.7)
	Yes	215 423 (5.2)	93 239 (9.8)	109 216 (8.5)	9 820 (0.8)	2 688 (0.5)	460 (0.3)

* For this variable, the 100% may be lower than the overall total due to missing records (150 missing records were identified).

**Table 2:** Characteristics associated with COVID-19 death, Peru 2020-2022, global bivariate analysis.

Characteristics		COVID-19 death		P
		No (n=3 905 346)	Yes (n=215 423)	
		n (%)	n (%)	
Dustrict altitude of residence				<0.001
	0 to 500 masl	2 715 947 (94.3)	162 850 (5.7)	
	50 to 2000 masl	336 325 (95.9)	14 448 (4.1)	
	2001 to 3500 masl	715 580 (96.0)	29 929 (4.0)	
	3501 masl and more	137 494 (94.4)	8 196 (5.6)	
Sex				<0.001
	Female	2 047 024 (96.3)	77 772 (3.7)	
	Male	1 858 322 (93.1)	137 651 (6.9)	
Age groups*				<0.001
	0 to 49 años	2 809 108 (99.0)	29 779 (1.0)	
	50 to 74 años	962 769 (89.3)	115 494 (10.7)	
	75 years and older	133 319 (65.5)	70 150 (34.5)	
Vaccination				<0.001
	No	1 985 976 (91.0)	197 262 (9.0)	
	1 dose	105 665 (95.3)	5 188 (4.7)	
	2 doses	807 569 (99.0)	8 170 (1.0)	
	3 or more doses	1 006 136 (99.5)	4 803 (0.5)	
Hospitalization				<0.001
	No	3 860 065 (95.6)	178 211 (4.4)	
	Yes	45 281 (54.9)	37 212 (45.1)	
Human development index				<0.001
	Low	565 693 (94.3)	34 312 (5.7)	
	Medium	1 755 012 (94.3)	106 722 (5.7)	
	High	1 086 538 (94.8)	59 485 (5.2)	
	Very high	498 103 (97.1)	14 904 (2.9)	
Population density				<0.001
	Low	679 439 (94.6)	38 667 (5.4)	
	Medium	621 740 (94.0)	39 383 (6.0)	
	High	2 604 167 (95.0)	137 373 (5.0)	

* For this variable, 100% may be lower than the global total due to a lack of records (150 missing records were identified

**Table 3:** Characteristics associated with COVID-19 death, Peru 2020-2022, bivariate analysis stratified by epidemic wave.

Characteristics		COVID-19 death (1st wave)		p	COVID-19 death (2nd wave)		p	COVID-19 death (3rd wave)		p	COVID-19 death (4th wave)		p	COVID-19 death (5th wave)		p
		No (n=859 572)	Yes (n=93 239)		No (n=1 171 565)	Yes (n=109 216)		No (n=1 161 665)	Yes (n=9 820)		No (n=564 630)	Yes (n=2 688)		No (n=147 914)	Yes (n=460)	
		n (%)	n (%)		n (%)	n (%)		n (%)	n (%)		n (%)	n (%)		n (%)	n (%)	
District altitude of residency				<0.001			<0.001			<0.001			<0.001			0.009
	0 a 500 masl	619 279 (89.3)	74 560 (10.7)		780 333 (90.8)	79 371 (9.2)		811 529 (99.2)	6 843 (0.8)		399 574 (99.6)	1 722 (0.4)		105 232 (99.7)	354 (0.3)	
	501 to 2000 masl	83 308 (93.7)	5 600 (6.3)		110 302 (93.4)	7 805 (6.6)		91 568 (99.2)	748 (0.8)		39 836 (99.4)	257 (0.6)		11 311 (99.7)	38 (0.3)	
	2001 to 3500 masl	125 632 (92.2)	10 641 (7.8)		235 666 (93.2)	17 122 (6.8)		217 770 (99.3)	1 565 (0.7)		109 618 (99.5)	544 (0.5)		26 894 (99.8)	57 (0.2)	
	3501 masl and more	31 353 (92.8)	2 438 (7.2)		45 264 (90.2)	4 918 (9.8)		40 798 (98.4)	664 (1.6)		15 602 (99.0)	165 (1.0)		4 477 (99.8)	11 (0.2)	
Sex				<0.001			<0.001			<0.001			<0.001			<0.001
	Female	427 038 (93.2)	31 115 (6.8)		592 023 (93.5)	41 025 (6.5)		623 348 (99.3)	4 267 (0.7)		320 412 (99.6)	1 164 (0.4)		84 203 (99.8)	201 (0.2)	
	Male	432 534 (87.4)	62 124 (12.6)		579 542 (89.5)	68 191 (10.5)		538 317 (99.0)	5 553 (1.0)		244 218 (99.4)	1 524 (0.6)		63 711 (99.6)	259 (0.4)	
Age group*				<0.001			<0.001			<0.001			<0.001			<0.001
	0 to 49 years	597 800 (98.0)	11 992 (2.0)		836 561 (98.2)	15 513 (1.8)		896 770 (99.8)	1 856 (0.2)		387 054 (99.9)	379 (0.1)		90 923 (∼100.0)	39 (∼0.0)	
	50 to 74 years	232 369 (81.9)	51 292 (18.1)		296 48 (83.2)	60 091 (16.9)		231 897 (98.6)	3 185 (1.4)		154 096 (99.5)	793 (0.5)		47 979 (99.7)	133 (0.3)	
	75 years and older	29 381 (49.5)	29 955 (50.5)		38 451 (53.4)	33 612 (46.6)		32 996 (87.3)	4 779 (12.7)		23 479 (93.9)	1 516 (6.1)		9 012 (96.9)	288 (3.1)	
Vaccination							<0.001			<0.001			<0.001			<0.001
	No	-	-	-	1 009 400 (90.9)	100 602 (9.1)		96 769 (96.9)	3 055 (3.1)		17 263 (98.1)	327 (1.9)		2 974 (98.7)	39 (1.3)	
	1 dose	-	-	-	44 180 (90.6)	4 585 (9.4)		45 736 (98.9)	524 (1.1)		13 622 (99.5)	71 (0.5)		2 127 (99.6)	8 (0.4)	
	2 doses	-	-	-	111 399 (96.6)	3 914 (3.4)		632 648 (99.4)	3 767 (0.6)		55 122 (99.2)	437 (0.8)		8 398 (99.4)	52 (0.6)	
	3 or more doses	-	-	-	6 586 (98.3)	115 (1.7)		386 512 (99.4)	2 474 (0.6)		478 623 (99.6)	1 853 (0.4)		134 415 (99.7)	361 (0.3)	
Hospitalization				<0.001			<0.001			<0.001			<0.001			<0.001
	No	843 861 (91.5)	78 813 (8.5)		1 146 907 (92.9)	88 105 (7.1)		1 158 066 (99.3)	8 406 (0.7)		563 593 (99.6)	2 454 (0.4)		147 638 (99.7)	433 (0.3)	
	Yes	15 711 (52.1)	14 426 (47.9)		24 658 (53.9)	21 111 (46.1)		3 599 (71.8)	1 414 (28.2)		1 037 (81.6)	234 (18.4)		276 (91.1)	27 (8.9)	
Human developmnet index				<0.001			<0.001			<0.001			<0.001			0.022
	Low	147 654 (92.0)	1 860 (8.0)		207 078 (91.7)	18 825 (8.3)		141 935 (98.6)	2 048 (1.4)		55 313 (99.1)	518 (0.9)		13 713 (99.6)	61 (0.4)	
	Medium	423 498 (89.8)	47 988 (10.2)		518 586 (90.8)	52 856 (9.2)		520 405 (99.2)	4 447 (0.8)		225 446 (99.5)	1 224 (0.5)		67 077 (99.7)	207 (0.3)	
	High	225 991 (89.3)	26 982 (10.7)		315 275 (91.5)	29 377 (8.5)		331 031 (99.3)	2 383 (0.7)		171 426 (99.6)	616 (0.4)		42 815 (99.7)	127 (0.3)	
	Very high	62 429 (92.0)	5 409 (8.0)		130 626 (94.1)	8 158 (5.9)		168 294 (99.4)	942 (0.6)		112 445 (99.7)	330 (0.3)		24 309 (99.7)	65 (0.3)	
Population density				<0.001			<0.001			<0.001			<0.001			0.009
	Low	178 715 (92.4)	14 649 (7.6)		228 496 (91.6)	21 019 (8.4)		179 339 (98.7)	2 304 (1.3)		72 610 (99.2)	618 (0.8)		20 279 (99.6)	77 (0.4)	
	Medium	148 489 (89.7)	17 097 (10.3)		193 746 (90.7)	19 964 (9.3)		182 887 (99.1)	1 739 (0.9)		74 095 (99.3)	497 (0.7)		22 523 (99.6)	89 (0.4)	
	High	532 368 (89.6)	61 493 (10.4)		749 323 (91.7)	68 233 (8.3)		799 439 (99.3)	5 777 (0.7)		417 925 (99.6)	1 573 (0.4)		105 112 (99.7)	460 (0.3)	

* For this variable, 100% may be lower than the global total due to a lack of records (150 missing records were identified).

In the multivariable analysis to evaluate the relationship between COVID-19 death and altitude (adjusted for sex, age group, vaccination, hospitalization, HDI, population density, and epidemic wave), a statistically significant association was evidenced. Compared with the altitude of 0-500 masl, residents of 501-2000 masl had a 32% (95%CI: 31% to 34%) lower risk of death and residents of 2001-3500 masl had a 31% (95%CI: 30% to 32%) lower risk of death, while residents of ≥3501 masl had no differences in the risk of death (p=0.498). These results can be seen in Table 4. Additionally, Table 5 shows the analysis performed by epidemic waves, where a similar trend can be evidenced in the adjusted multivariable models, consistently obtaining a lower risk of death in the 2001-3500 masl category compared to sea level in all epidemic waves.

**Table 4:** Association between district altitude of residence and COVID-19 death, Peru 2020-2022, global epidemiological approach.

Characteristics		Bivariate analysis		p	Multiple regression*✝		p
		RR	(95%CI)		RR	(95%CI)	
District altitude of residence							
	0 to 500 masl	Ref.		-	Ref.		-
	501 to 2000 masl	0.73	(0.62-0.85)	<0.001	0.67	(0.61-0.74)	<0.001
	2001 to 3500 masl	0.71	(0.63-0.80)	<0.001	0.68	(0.63-0.74)	<0.001
	3501 masl and more	0.99	(0.88-1.13)	0.932	0.98	(0.88-1.09)	0.666
Sex							
	Female	Ref.		-	Ref.		-
	Male	1.88	(1.85-1.91)	<0.001	1.52	(1.51-1.54)	<0.001
Age group^✝^							
	0 to 49 years	Ref.		-	Ref.		-
	50 to 74 years	10.21	(9.93-10.50)	<0.001	8.46	(8.21-8.71)	<0.001
	75 years and older	32.87	(32.43-34.38)	<0.001	24.45	(23.24-25.72)	<0.001
Vaccination							
	No	Ref.		-	Ref.		-
	1 dose	0.52	(0.46-0.58)	<0.001	0.71	(0.68-0.74)	<0.001
	2 doses	0.11	(0.10-0.12)	<0.001	0.35	(0.33-0.37)	<0.001
	3 or more doses	0.05	(0.05-0.06)	<0.001	0.15	(0.14-0.16)	<0.001
Hospitalization							
	No	Ref.		-	Ref.		-
	Yes	10.22	(9.61-10.88)	<0.001	3.29	(3.16-3.43)	<0.001
Human developmnet index							
	Low	Ref.		-	Ref.		-
	Medium	1.00	(0.91-1.11)	0.962	1.02	(0.94-1.11)	0.590
	High	0.91	(0.78-1.05)	0.198	0.90	(0.81-1.02)	0.091
	Very high	0.51	(0.40-0.64)	<0.001	0.67	(0.55-0.81)	<0.001
Population density							
	Low	Ref.		-	Ref.		-
	Medium	1.11	(0.97-1.26)	0.139	1.04	(0.94-1.15)	0.455
	High	0.93	(0.81-1.07)	0.301	1.13	(1.03-1.23)	0.007
Epidemic wave							
	1st wave	Ref.			Ref.		-
	2nd wave	0.87	(0.83-0.91)	<0.001	1.03	(0.99-1.07)	0.111
	3rd wave	0.09	(0.08-0.09)	<0.001	0.48	(0.44-0.52)	<0.001
	4th wave	0.05	(0.04-0.05)	<0.001	0.36	(0.32-0.40)	<0.001
	5th wave	0.03	(0.03-0.04)	<0.001	0.21	(0.18-0.24)	<0.001

*Poisson regression model with robust variances clustered by district, adjusted for individual variables (sex, age group, vaccination, hospitalization) and contextual variables (human development index, population density, epidemic wave).

✝ For this variable and model, 4,120,619 records were used.

RR: Relative risk; 95% CI: 95% confidence interval.

**Table 5:** Association between district altitude of residence and COVID-19 death, Peru 2020-2022, epidemiological approach by epidemic wave.

Characteristics		1st wave (n=952 811)						2nd wave (n= 1 280 781)						3rd wave (n=1 171 485)						4th wave (n=567 318)						5th wave (n=148 374)					
		Bivariate analysis		p	Multiple regression *✝		P	Bivariate analysis		p	Multiple regression **✝✝		p	Bivariate analysis		p	Multiple regression **✝✝✝		p	Bivariate analysis		p	Multiple regression **✝✝✝✝		p	Bivariate analysis		p	Multiple regression **		p
		RR	(95% CI)		RR	(95% CI)		RR	(95% CI)		RR	(95% CI)		RR	(95% CI)		RR	(95% CI)		RR	(95% CI)		RR	(95% CI)		RR	(95% CI)		RR	(95% CI)	
District altitude of residence																															
	0 to 500 masl	Ref.		-	Ref.		-	Ref.		-	Ref.		-	Ref.		-	Ref.		-	Ref.		-	Ref.		-	Ref.		-	Ref.		-
	0 to 500 masl	0.59	(0.52-0.67)	<0.001	0.65	(0.58-0.73)	<0.001	0.72	(0.61-0.84)	<0.001	0.68	(0.61-0.77)	<0.001	0.97	(0.80-1.17)	0.749	0.62	(0.53-0.72)	<0.001	1.49	(1.17-1.90)	0.001	0.86	(0.69-1.06)	0.156	1.00	(0.65-1.54)	0.995	0.76	(0.48-1.19)	0.233
	501 to 2000 masl	0.73	(0.65-0.81)	<0.001	0.72	(0.65-0.79)	<0.001	0.73	(0.65-0.82)	<0.001	0.66	(0.60-0.73)	<0.001	0.85	(0.71-1.03)	0.096	0.67	(0.60-0.74)	<0.001	1.15	(0.92-1.43)	0.210	0.67	(0.57-0.79)	<0.001	0.63	(0.47-0.85)	0.003	0.45	(0.34-0.60)	<0.001
	2001 to 3500 masl	0.67	(0.59-0.76)	<0.001	0.87	(0.79-0.97)	0.014	1.06	(0.95-1.18)	0.286	1.00	(0.87-1.14)	0.989	1.92	(1.50-2.44)	<0.001	1.01	(0.83-1.23)	0.933	2.44	(1.77-3.36)	<0.001	1.07	(0.77-1.48)	0.697	0.73	(0.43-1.25)	0.253	0.50	(0.29-0.88)	0.015
Sex																															
	Female	Ref.		-	Ref.		-	Ref.		-	Ref.		-	Ref.		-	Ref.		-	Ref.		-	Ref.		-	Ref.		-	Ref.		-
	Male	1.85	(1.81-1.89)	<0.001	1.59	(1.56-1.61)	<0.001	1.62	(1.59-1.66)	<0.001	1.48	(1.46-1.51)	<0.001	1.50	(1.43-1.58)	<0.001	1.42	(1.37-1.48)	<0.001	1.71	(1.59-1.85)	<0.001	1.59	(1.47-1.71)	<0.001	1.70	(1.45-2.00)	<0.001	1.57	(1.34-1.86)	<0.001
Age group																															
	0 to 49 years	Ref.		-	Ref.		-	Ref.		-	Ref.		-	Ref.		-	Ref.		-	Ref.		-	Ref.		-	Ref.		-	Ref.		-
	50 to 74 years	9.19	(8.92-9.48)	<0.001	8.29	(8.03-8.57)	<0.001	9.26	(8.94-9.58)	<0.001	8.28	(7.97-8.60)	<0.001	6.56	(6.14-7.01)	<0.001	8.57	(8.04-9.14)	<0.001	5.23	(4.56-6.00)	<0.001	6.43	(5.58-7.41)	<0.001	6.45	(4.50-9.23)	<0.001	7.17	(5.00-10.30)	<0.001
	75 years and older	25.67	(25.58-26.81)	<0.001	22.14	(21.16-23.16)	<0.001	25.62	(24.24-27.08)	<0.001	22.50	(21.20-23.77)	<0.001	61.25	(56.13-66.84)	<0.001	64.60	(58.26-71.64)	<0.001	62.00	(53.79-71.47)	<0.001	62.73	(54.28-72.49)	<0.001	72.23	(50.69-102.93)	<0.001	72.27	(50.32-103.80)	<0.001
Vaccination																															
	No	-		-	-		-	Ref.		-	Ref.		-	Ref.		-	Ref.		-	Ref.		-	Ref.		-	Ref.		-	Ref.		-
	1 dose	-		-	-		-	1.04	(0.98-1.10)	0.224	0.78	(0.75-0.81)	<0.001	0.37	(0.32-0.43)	<0.001	0.44	(0.39-0.49)	<0.001	0.28	(0.21-0.37)	<0.001	0.47	(0.37-0.60)	<0.001	0.29	(0.14-0.61)	0.001	0.49	(0.24-0.98)	0.044
	2 doses	-		-	-		-	0.37	(0.35-0.40)	<0.001	0.39	(0.37-0.41)	<0.001	0.19	(0.17-0.21)	<0.001	0.30	(0.27-0.32)	<0.001	0.42	(0.36-0.50)	<0.001	0.59	(0.51-0.68)	<0.001	0.48	(0.31-0.72)	<0.001	0.65	(0.43-1.00)	0.048
	3 or more doses	-		-	-		-	0.19	(0.16-0.23)	<0.001	0.14	(0.11-0.16)	<0.001	0.21	(0.19-0.23)	<0.001	0.13	(0.12-0.14)	<0.001	0.21	(0.18-0.24)	<0.001	0.22	(0.19-0.25)	<0.001	0.21	(0.15-0.29)	<0.001	0.24	(0.17-0.34)	<0.001
Hospitalization																															
	No	Ref.		-	Ref.		-	Ref.		-	Ref.		-	Ref.		-	Ref.		-	Ref.		-	Ref.		-	Ref.		-	Ref.		-
	Yes	5.60	(5.26-5.97)	<0.001	3.09	(2.96-3.23)	<0.001	6.47	(6.04-6.92)	<0.001	3.32	(3.15-3.49)	<0.001	39.14	(35.08-43.67)	<0.001	5.35	(4.87-5.89)	<0.001	42.47	(35.11-51.37)	<0.001	6.53	(5.47-7.79)	<0.001	30.47	(19.78-46.95)	<0.001	7.04	(4.19-11.84)	<0.001
Human development index																															
	Low	Ref.		-	Ref.		-	Ref.		-	Ref.		-	Ref.		-	Ref.		-	Ref.		-	Ref.		-	Ref.		-	Ref.		-
	Medium	1.27	(1.12-1.44)	<0.001	1.00	(0.91-1.11)	0.989	1.11	(1.01-1.22)	0.032	1.08	(0.97-1.19)	0.158	0.60	(0.53-0.67)	<0.001	0.87	(0.78-0.98)	0.019	0.58	(0.50-0.68)	<0.001	0.82	(0.68-0.98)	0.033	0.69	(0.51-0.94)	0.019	0.66	(0.48-0.90)	0.009
	High	1.33	(1.17-1.51)	<0.001	0.92	(0.81-1.05)	0.204	1.02	(0.88-1.19)	0.766	0.93	(0.80-1.07)	0.324	0.50	(0.41-0.61)	<0.001	0.74	(0.60-0.92)	0.006	0.39	(0.32-0.47)	<0.001	0.59	(0.45-0.76)	<0.001	0.67	(0.48-0.93)	0.018	0.58	(0.39-0.86)	0.006
	Very high	1.00	(0.76-1.30)	0.972	0.68	(0.56-0.83)	<0.001	0.71	(0.56-0.89)	0.004	0.70	(0.57-0.88)	0.002	0.39	(0.30-0.51)	<0.001	0.52	(0.40-0.69)	<0.001	0.32	(0.25-0.40)	<0.001	0.41	(0.30-0.55)	<0.001	0.60	(0.39-0.94)	0.024	0.41	(0.25-0.66)	<0.001
Population density																															
	Low	Ref.		-	Ref.		-	Ref.		-	Ref.		-	Ref.		-	Ref.		-	Ref.		-	Ref.		-	Ref.		-	Ref.		-
	Medium	1.36	(1.18-1.58)	<0.001	1.12	(0.99-1.26)	0.063	1.11	(0.98-1.26)	0.109	1.00	(0.89-1.12)	0.999	0.74	(0.62-0.89)	0.002	0.89	(0.78-1.00)	0.057	0.79	(0.65-0.96)	0.020	0.95	(0.78-1.16)	0.603	1.01	(0.72-1.41)	0.974	1.09	(0.75-1.58)	0.664
	High	1.37	(1.22-1.53)	<0.001	1.20	(1.08-1.34)	0.001	0.99	(0.88-1.12)	0.883	1.10	(0.98-1.23)	0.105	0.57	(0.48-0.67)	<0.001	0.95	(0.84-1.08)	0.474	0.44	(0.37-0.53)	<0.001	0.78	(0.64-0.95)	0.011	0.74	(0.56-1.00)	0.047	0.95	(0.65-1.39)	0.794

*Poisson regression model with robust variances clustered by district, adjusted for individual variables (sex, age group, hospitalization) and contextual variables (human development index, population density).

**Poisson regression model with robust variances clustered by district, adjusted for individual variables (sex, age group, vaccination, hospitalization) and contextual variables (human development index, population density).

✝ For this model, 952,789 records were used.

✝✝ For this model, 1,280,656 records were used.

✝✝✝ For this model, 1,171,483 records were used.

✝✝✝✝ For this model, 567,317 records were used.

RR: Relative risk; 95% CI: 95% confidence interval.

Finally, regarding the sensitivity analyses, for both the availability of health resources globally (Supplementary Material 2) and by epidemic waves (Supplementary Material 3), there were no substantial differences compared to what was found in the main analyses. Similarly, when evaluating the data including only deaths that were previously cases, both globally and by epidemic waves (Supplementary Material 4), no changes were evidenced in relation to the main reported association. It was evidenced that the difference was significant at all times when comparing the risk of death with the altitude of 0-500 masl, with the 2001-3500 masl category consistently presenting a lower risk of death. Likewise, the main results were also consistent when repeating the regressions using the alternative altitude classification, more aligned with the altitudinal floors proposed by Pulgar Vidal (Supplementary Material 5). All of this can be evidenced in the supplementary material.

## DISCUSSION

A lower risk of COVID-19 death was found in the intermediate and moderately high altitude categories when comparing against the 0-500 masl category, although in the evaluation by epidemic waves only the moderately high altitude category remained consistently associated, with no lower risk of death evidenced above 3500 masl.

The relationship between altitude and COVID-19 mortality has generated inconsistent results since the beginning of the pandemic. Initial studies in Latin America suggested a protective effect of high-altitude residence on mortality and infection ^10^^-^^12^, attributing the effect to chronic hypobaric hypoxia ^27^^-^^29^. However, these studies did not always adjust for relevant covariates nor consider the geographical asynchrony of the virus spread, especially considering that initial epicenters generally occurred in low-altitude areas.

Subsequent studies in Peru, using provincial data and ecological designs, did not find a monotonic protective trend. It was highlighted that differences in population density and other sociodemographic factors could partially explain the findings ^16^^,^^20^. International studies showed mixed results; for example, in the United States, case fatality decreased with altitude after adjusting for comorbidities and sociodemographic variables ^18^, while in Mexico, men above 2000 masl and women above 2500 masl presented a higher risk of death than inhabitants below 500 masl ^17^. This heterogeneity reflects the complexity of individual and contextual factors and suggests that the relationship between altitude and mortality might not be linear.

Recent research suggests that the relationship between altitude and mortality could follow a non-linear U-shaped pattern ^30^, where moderate altitudes favor protective adaptations against hypoxia, while extreme altitudes could induce chronic pathophysiological changes that increase mortality risk, such as pulmonary hypertension and cardiovascular alterations ^24^^,^^31^^,^^32^. Our study found evidence of a protective effect at intermediate altitudes, but not at extremely high altitudes, consistent with the hypothesis of a non-monotonic relationship. This pattern was maintained even when employing altitudinal classifications based on Pulgar Vidal's ecological floors, suggesting that the observed trend does not depend exclusively on the physiological cutoff points used.

The physiological plausibility of these findings is supported by mechanisms of adaptation to chronic hypoxia. It has been proposed that chronic hypoxia can influence the regulation of angiotensin-converting enzyme 2 (ACE2), which is the cellular entry receptor used by SARS-CoV-2 ^33^, and cardiovascular and respiratory physiology ^34^. However, this evidence is preliminary and the precise mechanisms linking altitude, hypoxia, and COVID-19 outcomes require specific experimental and clinical studies.

While the exact mechanism of the protective effect of altitude cannot be determined with certainty, it is known that chronic hypobaric hypoxia induces beneficial cardiovascular and respiratory adaptations. In contrast, acute administration of erythropoietin (EPO) in COVID-19 patients did not demonstrate efficacy ^35^, likely because these were non-adaptive and non-sustained interventions. Theoretically, controlled interventions that safely simulate hypoxia, such as altitude training or supervised exercise-induced hypoxia, could reproduce some adaptive advantages and benefit cardiovascular and respiratory health in people at various altitudes ^36^. Nevertheless, this idea is speculative and requires experimental studies to evaluate its safety and efficacy.

It is important to mention that avoiding infection and following biosafety measures are and will be the best way to guarantee one's integrity ^8^. Our findings must be interpreted considering the limitations inherent to the available data, including the lack of information on individual comorbidities, the heterogeneity in the availability of health resources, and the impossibility of considering district vaccination coverage. These limitations could introduce residual confounding and affect the validity and reliability of the results.

Because there is an undetermined number of asymptomatic cases, it is not possible to accurately estimate the risk of death over total infections, a limitation shared by all studies based on national registries. Furthermore, the use of secondary databases may have led to bias due to misclassification or incomplete registration of cases and deaths, especially in remote districts with less access to diagnostic resources. Additionally, during the first epidemic waves, diagnostic and epidemiological surveillance capacity evolved rapidly and unevenly across the territory, which could have generated differential detection of cases and deaths according to time and geographical location. If underreporting differed by altitude, in both confirmed cases and deaths, case fatality estimates could have been affected. Although the official MINSA database includes deaths that had not been previously registered as cases, the possibility of differential capture by altitude persists. Therefore, the results should be interpreted with caution.

The data on availability of health resources presented significant inconsistencies and extensive periods of time without reporting. Furthermore, given that the availability of hospitalization beds, ICU, IMCU beds, and emergency stretchers is a highly dynamic variable, it was decided to require specific correspondence between the district and the date of each case for its analytical incorporation. This methodological decision, aimed at reducing temporal measurement bias, increased the proportion of missing data and limited its use in the main analysis, such that only 42.7% of records had valid information. Consequently, these variables were not included in the main multivariable model and were evaluated only in sensitivity analyses, which are presented in the supplementary material.

Likewise, it was not possible to incorporate relevant individual comorbidities (such as diabetes, hypertension, or cancer) due to the nature of the study and the use of anonymized public databases that do not contain detailed clinical information. This limitation constitutes an important source of residual confounding, given that these comorbidities are strong predictors of COVID-19 mortality and could be differentially distributed according to altitude. Similarly, systematic and comparable information on population vaccination coverage at the district level and by epidemic period was not available, which prevented evaluating possible indirect community protection effects (herd effect). The absence of this variable could introduce additional contextual confounding, to the extent that the intensity of mass vaccination strategies could have varied between districts. Finally, other unmeasured factors, such as environmental pollution or uncaptured differences in health infrastructure, could be influencing the observed association. Nonetheless, all available and relevant individual and contextual variables for this association were included in the models to minimize residual confounding.

A limitation that should be taken into consideration in relation to the stratified analysis by epidemic wave is the use of national cutoff dates to define pandemic phases. The spread of SARS-CoV-2 in Peru was markedly asynchronous, with significant temporal differences between the Coast, Highland, and Jungle regions. In this context, stratification by national waves can introduce temporal confounding by comparing districts that were in different local epidemiological phases (onset, peak, or decline) within the same ország-level defined "epidemic wave." Consequently, part of the observed differences in case fatality could reflect variations in transmission intensity or pressure on the health system, rather than an intrinsic effect of altitude.

It is recognized that the data structure presents a natural hierarchy in which individuals are nested within residence districts, which introduces a correlation between observations within the same cluster (district). Therefore, the analysis was adjusted using a robust variance clustered by district, in order to explicitly correct intra-district dependence by adjusting the variance-covariance matrix taking into account the clustering structure inherent in the data design. However, it is highlighted that this approach adequately corrects variance for inference but does not model unobserved variability between districts, as a hierarchical or mixed-effects model would, which could affect the precision of confidence intervals and leave residual contextual confounding not captured by altitude-related factors (such as health system quality, territorial accessibility, public investment, social cohesion, among others).

The use of district altitude as a proxy for physiological exposure to chronic hypobaric hypoxia inherently implies a simplification. Although four ranges were categorized based on physiological thresholds described in the literature, this approach could introduce exposure misclassification bias. In particular, the ≥3500 masl category groups distinct ecological zones (Suni and Puna) whose levels of adaptation and physiological response to hypoxia may differ. The unification of both strata could attenuate real differences between them and bias estimates toward the null. This aspect was evaluated through an alternative sensitivity analysis using Pulgar Vidal's altitudinal classification. Likewise, the 0-500 masl category includes both coastal areas with high service availability and Lower Jungle zones with less healthcare provision. This structural heterogeneity could generate residual confounding not fully controllable, which is recognized as another relevant study limitation.

Another possible source of bias comes from the mobility of people who required care in centers located at different altitudes. It was decided to use the altitude of the place of residence instead of the place of death, given that the protective effect associated with chronic hypobaric hypoxia only occurs with sustained and prolonged exposures. Therefore, displacements to receive care would not generate a physiological protective effect, and even an acute transfer to higher altitude areas could represent a risk factor rather than a benefit. Even so, a measurement error derived from this mobility pattern cannot be completely ruled out. Among the strengths, it can be highlighted that this is the study covering the longest period of time for analysis in Peru. Furthermore, although altitude may vary within a single district, in most cases these differences do not exceed 1000 meters, a small magnitude compared to the more than 4000 meters observable between districts of the same province; therefore, the potential intra-district bias would be smaller in relation to the national altitudinal gradient.

In conclusion, in Peru, during the 2020-2022 period, the data do not support the hypothesis of a monotonic decrease in the risk of COVID-19 death with increasing altitude of residence. On the contrary, the risk consistently showed a non-linear U-shaped pattern, with lower case fatality among cases residing at intermediate altitudes (2001-3500 masl) in all performed analyses. These findings suggest that the role of altitude in COVID-19 case fatality is complex and likely conditioned by physiological and contextual processes, and reinforce the need to interpret geographical factors in conjunction with other structural determinants and with the sustained implementation of prevention and control measures in future epidemics.

It is recommended that future research continue exploring the possible non-linear relationship between altitude of residence and COVID-19 death, incorporating analytical approaches that allow for proper modeling of such non-linearity. Likewise, studies with individual and prospective designs should evaluate inflammatory, hematological markers, and other proteins related to the response to hypoxia to contribute to the understanding of the possible biological mechanisms underlying the differences observed according to geographical conditions.
