# Supplementary material for: Altitude and case fatality rate from COVID-19 during the pandemic in Peru, 2020-2022
Source: Rev Peru Med Exp Salud Publica. 2026 Mar 17;43(1):27–40. doi: 10.17843/rpmesp.2026.431.14979 (PMC13245995; doi:10.17843/rpmesp.2026.431.14979)
Supplement: Supplementary material. — Available in the electronic version of the RPMESP. [file rpmesp-43-01-14979-s001.docx]

| Material suplementario 1. Características de los pacientes con COVID-19 según altitud de residencia, Perú 2020-2022 (n=4 120 769) | | | | | |
| --- | --- | --- | --- | --- | --- |
| Características | | Altitud de residencia | | | |
|  |  | 0 a 500 msnm | 501 a 2000 msnm | 2001 a 3500 msnm | 3500 msnm a más |
|  |  | (n=2 878 797) | (n=350 773) | (n=745 509) | (n=145 690) |
| Sexo | | | | | |
|  | Femenino | 1 483 146 (51,5) | 179 867 (51,3) | 387 517 (52,0) | 74 266 (51,0) |
|  | Masculino | 1 395 651 (48,5) | 170 906 (48,7) | 357 992 (48,0) | 71 424 (49,0) |
|  | | | | | |
| Grupos de edad* | | | | | |
|  | 0 a 49 años | 1 967 545 (68,4) | 247 799 (70,7) | 522 101 (70,0) | 101 442 (69,6) |
|  | 50 a 74 años | 767 231 (26,7) | 87 386 (24,9) | 186 362 (25,0) | 37 284 (25,6) |
|  | 75 años a más | 143 890 (5,0) | 15 575 (4,4) | 37 043 (5,0) | 6 961 (4,8) |
|  | | | | | |
| Vacunación | | | | | |
|  | No | 1 511 938 (52,5) | 205 268 (58,5) | 381 528 (51,2) | 84 504 (58,0) |
|  | 1 dosis | 76 887 (2,7) | 9 293 (2,7) | 19 443 (2,6) | 5 230 (3,6) |
|  | 2 dosis | 559 922 (19,5) | 69 112 (19,7) | 155 098 (20,8) | 31 607 (21,7) |
|  | 3 o más dosis | 730 050 (25,4) | 67 100 (19,1) | 189 440 (25,4) | 24 349 (16,7) |
|  | | | | | |
| Hospitalización | | | | | |
|  | No | 2 829 857 (98,3) | 340 029 (96,9) | 725 387 (97,3) | 143 003 (98,2) |
|  | Si | 48 940 (1,7) | 10 744 (3,1) | 20 122 (2,7) | 2 687 (1,8) |
|  | | | | | |
| Índice de desarrollo humano | | | | | |
|  | Bajo | 203 413 (7,1) | 117 871 (33,6) | 212 388 (28,5) | 66 333 (45,5) |
|  | Medio | 1 177 517 (40,9) | 185 789 (53,0) | 419 981 (56,3) | 78 447 (53,9) |
|  | Alto | 984 860 (34,2) | 47 113 (13,4) | 113 140 (15,18) | 910 (0,6) |
|  | Muy alto | 513 007 (17,8) | 0 (0,0) | 0 (0,0) | 0 (0,0) |
|  | | | | | |
| Densidad poblacional | | | | | |
|  | Baja | 268 437 (9,3) | 220 910 (63,0) | 146 187 (19,6) | 82 572 (56,7) |
|  | Media | 410 501 (14,3) | 49 904 (14,2) | 142 243 (19,1) | 58 475 (40,1) |
|  | Alta | 2 199 859 (76,4) | 79 959 (22,8) | 457 079 (61,3) | 4 643 (3,2) |
|  | | | | | |
| Muerte por COVID-19 | | | | | |
|  | No | 2 715 947 (94,3) | 336 325 (95,9) | 715 580 (96,0) | 137 494 (94,4) |
|  | Si | 162 850 (5,7) | 14 448 (4,1) | 29 929 (4,0) | 8 196 (5,6) |
| * Para esta variable el 100% puede ser menor que el global por falta de registros (se contó con 150 registros ausentes). | | | | | |

**MATERIAL SUPLEMENTARIO**

| Material suplementario 2. Asociación entre altitud distrital de residencia y la muerte por COVID-19, Perú 2020-2022, análisis de sensibilidad con recursos de salud disponibles | | | | | | | | | | | | | | | | | | | | | | | | |
| --- | --- | --- | --- | --- | --- | --- | --- | --- | --- | --- | --- | --- | --- | --- | --- | --- | --- | --- | --- | --- | --- | --- | --- | --- |
| Características | | Regresión final*  (n = 4 120 619) | | p |  | Análisis con camas de hospitalización**  (n = 1 762 552) | | p |  | Análisis con camas de cuidados intensivos***  (n = 1 762 552) | | p |  | Análisis con camas de cuidados intermedios****  (n = 1 762 552) | | p |  | Análisis con camillas de emergencia*****  (n = 1 762 552) | | p |  | Análisis con todos los recursos de salud******  (n = 1 762 552) | | p |
|  |  | RR | (IC 95%) |  |  | RR | (IC 95%) |  |  | RR | (IC 95%) |  |  | RR | (IC 95%) |  |  | RR | (IC 95%) |  |  | RR | (IC 95%) |  |
| Altitud distrital de residencia | | | | |  |  |  |  |  |  |  |  |  |  |  |  |  |  |  |  |  |  |  |  |
|  | 0 a 500 msnm | Ref. | | - |  | Ref. | | - |  | Ref. | | - |  | Ref. | | - |  | Ref. | | - |  | Ref. | | - |
|  | 501 a 2000 msnm | 0,67 | (0,61-0,74) | <0,001 |  | 0,64 | (0,53-0,78) | <0,001 |  | 0,64 | (0,53-0,77) | <0,001 |  | 0,64 | (0,53-0,78) | <0,001 |  | 0,64 | (0,53-0,78) | <0,001 |  | 0,64 | (0,53-0,78) | <0,001 |
|  | 2001 a 3500 msnm | 0,68 | (0,63-0,74) | <0,001 |  | 0,58 | (0,52-0,65) | <0,001 |  | 0,59 | (0,53-0,66) | <0,001 |  | 0,58 | (0,52-0,65) | <0,001 |  | 0,59 | (0,52-0,66) | <0,001 |  | 0,59 | (0,52-0,66) | <0,001 |
|  | 3501 msnm a más | 0,98 | (0,88-1,09) | 0,666 |  | 1,01 | (0,79-1,29) | 0,933 |  | 1,01 | (0,79-1,29) | 0,959 |  | 1,01 | (0,79-1,29) | 0,930 |  | 1,02 | (0,80-1,30) | 0,894 |  | 1,00 | (0,79-1,28) | 0,979 |
| *Modelo de regresión de Poisson con varianzas robustas agrupadas por distrito, ajustado por variables individuales (sexo, grupo de edad, vacunación, hospitalización) y contextuales (índice de desarrollo humano, densidad poblacional, ola epidémica). | | | | | | | | | | | | | | | | | | | | | | | | |
| **Modelo de regresión de Poisson con varianzas robustas agrupadas por distrito, ajustado por variables individuales (sexo, grupo de edad, vacunación, hospitalización) y contextuales (índice de desarrollo humano, densidad poblacional, ola epidémica y camas de hospitalización disponibles). | | | | | | | | | | | | | | | | | | | | | | | | |
| ***Modelo de regresión de Poisson con varianzas robustas agrupadas por distrito, ajustado por variables individuales (sexo, grupo de edad, vacunación, hospitalización) y contextuales (índice de desarrollo humano, densidad poblacional, ola epidémica y camas de cuidados intensivos disponibles). | | | | | | | | | | | | | | | | | | | | | | | | |
| ****Modelo de regresión de Poisson con varianzas robustas agrupadas por distrito, ajustado por variables individuales (sexo, grupo de edad, vacunación, hospitalización) y contextuales (índice de desarrollo humano, densidad poblacional, ola epidémica y camas de cuidados intermedios disponibles). | | | | | | | | | | | | | | | | | | | | | | | | |
| *****Modelo de regresión de Poisson con varianzas robustas agrupadas por distrito, ajustado por variables individuales (sexo, grupo de edad, vacunación, hospitalización) y contextuales (índice de desarrollo humano, densidad poblacional, ola epidémica y camillas de emergencia disponibles). | | | | | | | | | | | | | | | | | | | | | | | | |
| ******Modelo de regresión de Poisson con varianzas robustas agrupadas por distrito, ajustado por variables individuales (sexo, grupo de edad, vacunación, hospitalización) y contextuales (índice de desarrollo humano, densidad poblacional, ola epidémica y camas de hospitalización, cuidados intensivos, cuidados intermedios y camillas de emergencia disponibles). | | | | | | | | | | | | | | | | | | | | | | | | |
| RR: Riesgo relativo; IC 95%: Intervalo de confianza al 95%. | | | | | | | | | | | | | | | | | | | | | | | | |

| Material suplementario 3. Asociación entre altitud distrital de residencia y la muerte por COVID-19, Perú 2020-2022, análisis de sensibilidad con recursos de salud disponibles por olas epidémicas | | | | | | | | | | | | | | | | | | | | | | | | |
| --- | --- | --- | --- | --- | --- | --- | --- | --- | --- | --- | --- | --- | --- | --- | --- | --- | --- | --- | --- | --- | --- | --- | --- | --- |
| Características | | Regresión final para la  2da ola*  (n = 1 280 656) | | p |  | Análisis con los recursos de salud para la 2da ola**  (n = 519 157) | | p |  | Regresión final para la  3ra ola*  (n = 1 171 483) | | p |  | Análisis con los recursos de salud para la 3ra ola**  (n = 891 267) | | p |  | Regresión final para la  4ta ola*  (n = 567 317) | | p |  | Análisis con los recursos de salud para la 4ta ola**  (n = 352 128) | | p |
|  |  | RR | (IC 95%) |  |  | RR | (IC 95%) |  |  | RR | (IC 95%) |  |  | RR | (IC 95%) |  |  | RR | (IC 95%) |  |  | RR | (IC 95%) |  |
| Altitud distrital de residencia | | | | |  |  | | |  |  | | |  |  | | |  |  | | |  |  | | |
|  | 0 a 500 msnm | Ref. | | - |  | Ref. | | - |  | Ref. | | - |  | Ref. | | - |  | Ref. | | - |  | Ref. | | - |
|  | 501 a 2000 msnm | 0,68 | (0,61-0,77) | <0,001 |  | 0,61 | (0,49-0,76) | <0,001 |  | 0,62 | (0,53-0,72) | <0,001 |  | 0,71 | (0,57-0,88) | 0,002 |  | 0,86 | (0,69-1,06) | 0,156 |  | 0,80 | (0,57-1,13) | 0,202 |
|  | 2001 a 3500 msnm | 0,66 | (0,60-0,73) | <0,001 |  | 0,58 | (0,52-0,65) | <0,001 |  | 0,67 | (0,60-0,74) | <0,001 |  | 0,58 | (0,49-0,69) | <0,001 |  | 0,67 | (0,57-0,79) | <0,001 |  | 0,57 | (0,46-0,70) | <0,001 |
|  | 3501 msnm a más | 1,00 | (0,87-1,14) | 0,989 |  | 0,96 | (0,76-1,21) | 0,727 |  | 1,01 | (0,83-1,23) | 0,933 |  | 1,09 | (0,76-1,56) | 0,644 |  | 1,07 | (0,77-1,48) | 0,697 |  | 1,28 | (0,71-2,28) | 0,411 |
| *Modelo de regresión de Poisson con varianzas robustas agrupadas por distrito, ajustado por variables individuales (sexo, grupo de edad, vacunación, hospitalización) y contextuales (índice de desarrollo humano, densidad poblacional). | | | | | | | | | | | | | | | | | | | | | | | | |
| **Modelo de regresión de Poisson con varianzas robustas agrupadas por distrito, ajustado por variables individuales (sexo, grupo de edad, vacunación, hospitalización) y contextuales (índice de desarrollo humano, densidad poblacional y camas de hospitalización, cuidados intensivos, cuidados intermedios y camillas de emergencia disponibles). | | | | | | | | | | | | | | | | | | | | | | | | |
| RR: Riesgo relativo; IC 95%: Intervalo de confianza al 95%. | | | | | | | | | | | | | | | | | | | | | | | | |

| Material suplementario 4. Asociación entre altitud distrital de residencia y la muerte por COVID-19, Perú 2020-2022, análisis de sensibilidad con las muertes de casos confirmados: total y según ola epidémica | | | | | | | | | | | | | | | | | | | | | | | | |
| --- | --- | --- | --- | --- | --- | --- | --- | --- | --- | --- | --- | --- | --- | --- | --- | --- | --- | --- | --- | --- | --- | --- | --- | --- |
| Características | | Total (n=4 013 694) | | |  | 1ra ola (n=905 215) | | |  | 2da ola (n=1 224 914) | | |  | 3ra ola (n=1 168 805) | | |  | 4ta ola (n=566 502) | | |  | 5ta ola (n=148 258) | | |
|  |  | Regresión múltiple* | | p |  | Regresión múltiple** | | p |  | Regresión múltiple*** | | p |  | Regresión múltiple*** | | p |  | Regresión múltiple*** | | p |  | Regresión múltiple*** | | p |
|  |  | RR | (IC 95%) |  |  | RR | (IC 95%) |  |  | RR | (IC 95%) |  |  | RR | (IC 95%) |  |  | RR | (IC 95%) |  |  | RR | (IC 95%) |  |
| Altitud distrital de residencia | | | | | | | | | | | | | | | | | | | | | | | | |
|  | 0 a 500 msnm | Ref. | | - |  | Ref. | | - |  | Ref. | | - |  | Ref. | | - |  | Ref. | | - |  | Ref. | | - |
|  | 501 a 2000 msnm | 0.74 | (0,67-0,82) | <0,001 |  | 0,74 | (0,64-0,85) | <0,001 |  | 0,74 | (0,67-0,82) | <0,001 |  | 0,63 | (0,54-0,74) | <0,001 |  | 0,88 | (0,71-1,08) | 0,223 |  | 0,76 | (0,46-1,24) | 0,270 |
|  | 2001 a 3500 msnm | 0.71 | (0,64-0,79) | <0,001 |  | 0,73 | (0,66-0,82) | <0,001 |  | 0,69 | (0,61-0,78) | <0,001 |  | 0,68 | (0,61-0,77) | <0,001 |  | 0,75 | (0,62-0,89) | 0,001 |  | 0,45 | (0,31-0,64) | <0,001 |
|  | 3501 msnm a más | 1.02 | (0,94-1,11) | 0,585 |  | 0,90 | (0,81-0,99) | 0,034 |  | 1,04 | (0,92-1,17) | 0,549 |  | 0,97 | (0,83-1,14) | 0,730 |  | 1,13 | (0,81-1,58) | 0,483 |  | 0,59 | (0,33-1,07) | 0,081 |
| *Modelo de regresión de Poisson con varianzas robustas agrupadas por distrito, ajustado por variables individuales (sexo, grupo de edad, vacunación, hospitalización) y contextuales (índice de desarrollo humano, densidad poblacional, ola epidémica). | | | | | | | | | | | | | | | | | | | | | | | | |
| **Modelo de regresión de Poisson con varianzas robustas agrupadas por distrito, ajustado por variables individuales (sexo, grupo de edad, hospitalización) y contextuales (índice de desarrollo humano, densidad poblacional). | | | | | | | | | | | | | | | | | | | | | | | | |
| ***Modelo de regresión de Poisson con varianzas robustas agrupadas por distrito, ajustado por variables individuales (sexo, grupo de edad, vacunación, hospitalización) y contextuales (índice de desarrollo humano, densidad poblacional). | | | | | | | | | | | | | | | | | | | | | | | | |

Nota: Dada la escasa cantidad de registros y variabilidad de resultado en la categoría de 4001 msnm a más para la 5ta ola epidémica, la comparación con dicha categoría no fue posible, por lo que se omitió dicha información en la tabla.

| Material suplementario 5. Asociación entre altitud distrital de residencia y la muerte por COVID-19, Perú 2020-2022, análisis de sensibilidad con las categorías de altitud según la clasificación de Pulgar Vidal | | | | | | | | | | | | | | | | | | | | | | | | |
| --- | --- | --- | --- | --- | --- | --- | --- | --- | --- | --- | --- | --- | --- | --- | --- | --- | --- | --- | --- | --- | --- | --- | --- | --- |
| Características | | Total (n=4 120 619) | | |  | 1ra ola (n=952 789) | | |  | 2da ola (n=1 280 656) | | |  | 3ra ola (n=1 171 483) | | |  | 4ta ola (n=567 317) | | |  | 5ta ola (n=148 374) | | |
|  |  | Regresión múltiple* | | p |  | Regresión múltiple** | | p |  | Regresión múltiple*** | | p |  | Regresión múltiple*** | | p |  | Regresión múltiple*** | | p |  | Regresión múltiple*** | | p |
|  |  | RR | (IC 95%) |  |  | RR | (IC 95%) |  |  | RR | (IC 95%) |  |  | RR | (IC 95%) |  |  | RR | (IC 95%) |  |  | RR | (IC 95%) |  |
| Altitud distrital de residencia | | | | | | | | | | | | | | | | | | | | | | | | |
|  | 0 a 500 msnm | Ref. | | - |  | Ref. | | - |  | Ref. | | - |  | Ref. | | - |  | Ref. | | - |  | Ref. | | - |
|  | 501 a 2300 msnm | 0.67 | (0,61-0,74) | <0,001 |  | 0,65 | (0,58-0,73) | <0,001 |  | 0,68 | (0,61-0,76) | <0,001 |  | 0,61 | (0,53-0,70) | <0,001 |  | 0,85 | (0,69-1,04) | 0,122 |  | 0,75 | (0,48-1,17) | 0,199 |
|  | 2301 a 3500 msnm | 0.68 | (0,63-0,74) | <0,001 |  | 0,72 | (0,65-0,79) | <0,001 |  | 0,66 | (0,60-0,73) | <0,001 |  | 0,67 | (0,60-0,75) | <0,001 |  | 0,67 | (0,57-0,78) | <0,001 |  | 0,44 | (0,33-0,59) | <0,001 |
|  | 3501 a 4000 msnm | 0,97 | (0,86-1,10) | 0,659 |  | 0,89 | (0,79-0,99) | 0,036 |  | 0,99 | (0,85-1,14) | 0,854 |  | 1,00 | (0,81-1,24) | 0,988 |  | 1,05 | (0,74-1,49) | 0,791 |  | 0,59 | (0,34-1,03) | 0,061 |
|  | 4001 msnm a más | 1.00 | (0,89-1,11) | 0,957 |  | 0,80 | (0,69-0,92) | 0,002 |  | 1,11 | (0,97-1,27) | 0,133 |  | 1,05 | (0,80-1,36) | 0,736 |  | 1,27 | (0,70-2,29) | 0,437 |  | - | - | - |
| *Modelo de regresión de Poisson con varianzas robustas agrupadas por distrito, ajustado por variables individuales (sexo, grupo de edad, vacunación, hospitalización) y contextuales (índice de desarrollo humano, densidad poblacional, ola epidémica). | | | | | | | | | | | | | | | | | | | | | | | | |
| **Modelo de regresión de Poisson con varianzas robustas agrupadas por distrito, ajustado por variables individuales (sexo, grupo de edad, hospitalización) y contextuales (índice de desarrollo humano, densidad poblacional). | | | | | | | | | | | | | | | | | | | | | | | | |
| ***Modelo de regresión de Poisson con varianzas robustas agrupadas por distrito, ajustado por variables individuales (sexo, grupo de edad, vacunación, hospitalización) y contextuales (índice de desarrollo humano, densidad poblacional). | | | | | | | | | | | | | | | | | | | | | | | | |
