# Supplementary material for: Altitude and case fatality rate from COVID-19 during the pandemic in Peru, 2020-2022
Source: Rev Peru Med Exp Salud Publica. 2026 Mar 17;43(1):27–40. doi: 10.17843/rpmesp.2026.431.14979 (PMC13245995; doi:10.17843/rpmesp.2026.431.14979)
Supplement: Translation

# Translation: Altitud y letalidad por COVID-19 durante la pandemia en el Perú, 2020-2022

**Authors:** Rodrigo Robles-Mariños, Diego Fano-Sizgorich, Germán F. Alvarado, Gustavo F. Gonzales

**Affiliations:** 1 Facultad de Salud Pública y Administración, Universidad Peruana Cayetano Heredia, Lima, Perú.; 2 Laboratorio de Endocrinología y Reproducción, Facultad de Ciencias e Ingeniería, Departamento de Ciencias Biológicas y Fisiológicas, Universidad Peruana Cayetano Heredia, Lima, Perú.; 3 Facultad de Ciencias de la Salud, Universidad San Ignacio de Loyola, Lima, Perú.

**Keywords:** COVID-19, Letalidad, Mortalidad, Altitud, Perú

## Abstract

**Objetivos.:**

Evaluar la forma funcional de la asociación entre la altitud distrital de residencia y la letalidad de los casos de la COVID-19 en Perú durante el periodo 2020-2022.

**Materiales y métodos.:**

Se realizó un estudio de cohorte retrospectivo utilizando datos secundarios del Ministerio de Salud del Perú sobre casos y muertes por COVID-19. La altitud distrital de residencia se categorizó en cuatro grupos (0-500; 501-2000; 2001-3500; y ≥3501 msnm). El desenlace fue la muerte por COVID-19 según el registro oficial de defunciones. Se empleó regresión de Poisson para estimar riesgos relativos ajustados por variables individuales y contextuales, utilizando varianza robusta agrupada por distrito. Los análisis se repitieron según las cinco olas epidémicas ocurridas entre marzo de 2020 y diciembre de 2022.

**Resultados.:**

Se observó una asociación no lineal entre la altitud de residencia y la muerte por COVID-19, compatible con un patrón en forma de “U”, caracterizada por un menor riesgo de muerte en los casos residentes entre 2001 y 3500 msnm en comparación con aquellos que residían entre 0 y 500 msnm. Este patrón se mantuvo de forma consistente en los análisis estratificados por ola epidémica.

**Conclusiones.:**

En Perú, durante el periodo 2020-2022, los datos no respaldan una disminución monotónica del riesgo de muerte por COVID-19 con el aumento de la altitud de residencia. Por el contrario, los resultados sugieren una relación no lineal, con menor letalidad en altitudes intermedias (2001-3500 msnm).

## INTRODUCCIÓN

Perú reportó sus primeros casos de la enfermedad por coronavirus 2019 (COVID-19) en marzo de 2020, y en las semanas siguientes rápidamente aumentaron los casos y muertes en muchas regiones. Inicialmente, el criterio para definir una muerte por la COVID-19 se basó en la existencia de una prueba positiva previo al fallecimiento ^1^. Sin embargo, muchas muertes atribuibles a la COVID-19 no estaban siendo registradas como tal. Para subsanar esta subestimación, el Ministerio de Salud del Perú (MINSA) estableció siete criterios clínico-epidemiológicos más sensibles para el registro de defunción por COVID-19, lo que casi triplicó el número de muertes contabilizadas ^(^^2^.

La mortalidad y letalidad por la COVID-19 varía según características individuales. Adultos mayores y varones presentan mayor riesgo de muerte ^3^^,^^4^, al igual que personas con comorbilidades como cáncer, enfermedad coronaria, hipertensión, obesidad, diabetes, enfermedades respiratorias y renales ^5^^,^^6^. Sin embargo, la mortalidad también depende de factores contextuales, como las medidas de bioseguridad implementadas y la capacidad de los sistemas de atención médica ^(^^7^^,^^8^. La introducción de las vacunas constituyó una estrategia clave para reducir la mortalidad y letalidad ^(^^9^. La consideración simultánea de factores individuales y contextuales es esencial para comprender los determinantes de la letalidad por COVID-19, ya que enfocarse únicamente en uno de estos niveles podría llevar a conclusiones parciales o sesgadas.

La altitud ha sido identificada como un factor potencialmente relacionado con la mortalidad por COVID-19, aunque la evidencia es contradictoria. Estudios iniciales en Bolivia y Ecuador sugirieron un efecto protector de la altitud tanto sobre el contagio como sobre la muerte ^10^, y hallazgos similares se reportaron en otros análisis internacionales ^11^^,^^12^. Sin embargo, investigaciones posteriores no encontraron asociación ^13^^-^^16^ o incluso reportaron un mayor riesgo de muerte a altitudes elevadas ^(^^17^. Ajustes por comorbilidades y variables sociodemográficas indicaron una disminución en la letalidad con la altitud en algunos estudios ^18^. Estas discrepancias reflejan la complejidad del fenómeno y la necesidad de estudios rigurosos que consideren múltiples factores simultáneamente ^19^^,^^20^.

En Perú, país con gran variación altitudinal, resulta fundamental evaluar la relación entre altitud y letalidad por COVID-19 con un enfoque más detallado. Estudios previos han utilizado la altitud a nivel provincial o diseños exclusivamente ecológicos ^16^^,^^20^. Este estudio intenta superar esas limitaciones al integrar un enfoque que combina factores individuales (sexo, edad, vacunación, hospitalización) y contextuales (altitud distrital, densidad poblacional, índice de desarrollo humano), lo que constituye una aproximación metodológica más completa y robusta.

El objetivo principal del presente estudio fue evaluar la forma funcional de la asociación entre la altitud distrital de residencia y la letalidad de los casos de COVID-19 en Perú durante el periodo 2020-2022. Como objetivo secundario, se planteó analizar dicha asociación según las cinco olas epidémicas ocurridas en el país, considerando la variación en la capacidad de respuesta sanitaria y el impacto de las estrategias de vacunación, ambos factores influenciados por la aparición de diferentes variantes del virus a lo largo de la pandemia.

**MENSAJE CLAVE**

Motivación para realizar el estudio. La relación entre la muerte por COVID-19 y la altitud es controversial. En Perú, país con gran variación altitudinal, explorar esta relación es clave para desarrollar estrategias de prevención y control de este tipo de enfermedades.

Principales hallazgos. Se encontró una posible asociación en forma de “U”, donde los casos que residían entre 2001-3500 msnm tuvieron un menor riesgo de muerte por COVID-19.

Implicancias para la salud pública. Estos hallazgos destacan la necesidad de un enfoque integral que considere factores geográficos junto con medidas preventivas efectivas para hacer frente a epidemias de esta naturaleza.

## MATERIALES Y MÉTODOS

### Diseño de estudio

Se realizó un estudio de cohorte retrospectivo, mediante bases de datos de acceso abierto. El periodo de evaluación fue de marzo del 2020 a diciembre del 2022.

### Población de estudio

La población de estudio estuvo compuesta por los casos de la COVID-19 registrados en la base del MINSA. Como unidad de análisis se consideró a cada caso de forma individual. Las muertes por la COVID-19 sin pruebas positivas también fueron incluidas en el estudio. Se tomó en cuenta el periodo de ±90 días para considerar un nuevo episodio de la COVID-19. Para las defunciones no reportadas como casos previamente, se tomó la fecha de defunción como fecha de caso. Se excluyeron los registros incompletos o inconsistentes que no permitieron enlazar los datos de muerte y altitud.

### Variables

La muerte por la COVID-19 fue la variable dependiente principal, y se apoyó en el registro en la base del MINSA, la cual consigna las muertes según cualquiera de los siguientes siete criterios ^2^:

1) virológico: presencia de una prueba virológica positiva en los 60 días previos; 

2) serológico: una prueba serológica positiva IgM o IgM/IgG en los 60 días previos; 

3) radiológico: muerte de un caso probable con evidencia radiológica, tomográfica o resonancia magnética nuclear compatible con una neumonía por la COVID-19; 

4) nexo epidemiológico: muerte de un caso probable que presenta nexo epidemiológico con un caso confirmado de la COVID-19; 

5) investigación epidemiológica: muerte de un caso sospechoso que es verificado por una investigación epidemiológica; 

6) clínico: muerte de un caso sospechoso que presenta cuadro clínico compatible con la COVID-19; y 

7) SINADEF (Sistema Informático Nacional de Defunciones): evidencia del certificado de defunción en el que se presenta el diagnóstico de la COVID-19 como causa de muerte.

Para fines del estudio, la variable sólo consideró si el caso estaba presente en dicha base de datos, independientemente del criterio empleado para su inclusión.

La variable de exposición principal fue la altitud distrital de residencia y se categorizó según las variaciones fisiológicas reportadas en la literatura, optando por la generación de cuatro categorías: 0-500 msnm (altitud baja), 501-2000 msnm (altitud intermedia), 2001-3500 msnm (altitud moderadamente alta) y ≥3501 msnm (altitud muy alta). Esta clasificación se basó en el primer piso altitudinal, el cual se relacionó con el nivel del mar (0-500 msnm) ^21^, luego se estableció como segundo punto de corte a los 2000 (msnm), punto utilizado para establecer una altitud moderada/alta relacionada con cambios fisiológicos ^22^^,^^23^^)^ en posible relación con el efecto protector de la altitud. Finalmente, se estableció el punto de corte a los 3500 msnm, ya que a partir de esta altitud se considera una altitud muy alta, relacionada con una mala adaptación y cambios patológicos incluso en aparentemente sanos ^(^^24^.

Si bien para el análisis principal se categorizó a la altitud en los cuatro rangos mencionados basados en variaciones fisiológicas descritas en la literatura, reconocemos que esta categorización implica una simplificación de una exposición continua y que no se corresponde exactamente con los pisos altitudinales bioclimáticos del Perú (por ejemplo, según la clasificación de Pulgar Vidal) ^25^. Esta decisión metodológica se consideró adecuada para el análisis principal por alinearse con los puntos de inflexión reportados en la respuesta fisiológica a la hipoxia. No obstante, se reconoce la posibilidad de mala clasificación no diferencial de la exposición. Por ende, como parte de un análisis de sensibilidad, se realizó una recategorización alternativa de la altitud más alineada con los pisos altitudinales propuestos por Pulgar Vidal, con el fin de evaluar la robustez del patrón observado bajo un esquema ecológico tradicionalmente aplicado al contexto peruano. De esta forma, se generaron las siguientes categorías: 0-500 msnm, 501-2300 msnm, 2301-3500 msnm, 3501-4000 msnm y ≥4001 msnm.

Con respecto a las covariables a nivel individual, se tomó en cuenta el sexo, reportado como masculino o femenino. Se categorizó a la edad en grupos según el riesgo de muerte por la COVID-19 en base a los reportes de los Centros para el Control y la Prevención de Enfermedades (CDC), estableciéndose las categorías: 0-49 años, 50-74 años y ≥75 años. La vacunación contra la COVID-19 se evaluó tomando en cuenta el registro de las dosis de inmunización a la fecha de caso, generándose las categorías de “no vacunado”, “1 dosis” (esquema incompleto), “2 dosis” (esquema completo) y “3 o más dosis” (esquema completo + dosis de refuerzo). Además, se trabajó con el dato de hospitalización por la COVID-19 como un proxy de severidad de la infección, y se tomó un periodo de tiempo de ±90 días de la fecha de reporte como caso para considerar la hospitalización como parte del episodio reportado.

En lo que respecta a las covariables a nivel distrital residencial, se obtuvo al índice de desarrollo humano (IDH), la densidad poblacional y la disponibilidad de recursos sanitarios. Respecto al IDH, el dato fue atribuido tomando en cuenta el reporte del Instituto Peruano de Economía para cada distrito. Luego, según las categorías propuestas por las Naciones Unidas, se generó la clasificación: IDH bajo (<55,0%), IDH medio (55,0-69,9%), IDH alto (70,0-79,9%) e IDH muy alto (>80,0%). La densidad poblacional se calculó dividiendo a la población total estimada por distrito según el Instituto Nacional de Estadística e Informática (INEI) para el 2020 entre la superficie distrital (en km^2^). Luego se categorizó según las categorías propuestas en un estudio que evaluó la eficiencia del sistema de salud en prevención y tratamiento frente a la COVID-19 ^26^: densidad baja (≤100 hab/km^2^), densidad media (>100 y ≤500 hab/km^2^) y densidad alta (>500 hab/km^2^). La disponibilidad de recursos sanitarios estuvo compuesta por la disponibilidad de camas de hospitalización, de unidad de cuidados intensivos (UCI), de unidad de cuidados intermedios (UCIN) y de camillas de emergencia. Estos datos fueron obtenidos según el registro de la Superintendencia Nacional de Salud (SUSALUD) del Perú, quienes brindaron un registro de la disponibilidad de dichos recursos por cada establecimiento de salud, aunque no se contó con un registro completo para todo el estudio.

Finalmente, se generó la variable de ola epidémica considerando como punto de corte el inicio de cada una de las 5 olas epidémicas ocurridas en Perú hasta diciembre de 2022, según las fechas declaradas por el Centro Nacional de Epidemiología, Prevención y Control de Enfermedades (CDC-Perú) y el MINSA, publicadas en salas situacionales de la COVID-19 y en el diario oficial del Perú, El Peruano. Las fechas contempladas como inicio para cada ola epidémica fueron: 06/03/2020 para la 1ra; 30/11/2020 para la 2da; 04/01/2022 para la 3ra; 27/06/2022 para la 4ta; y 02/12/2022 para la 5ta ola epidémica. Reconocemos que la propagación del SARS-CoV-2 en el territorio peruano fue geográficamente heterogénea y asincrónica, y que estas fechas no reflejan necesariamente el inicio o el pico epidémico local en cada distrito. Sin embargo, no existen definiciones estandarizadas de olas epidémicas a nivel regional o distrital para todo el periodo de estudio, por lo que se optó por esta aproximación con fines descriptivos y de estratificación temporal.

### Recolección de datos

Las bases de datos (xls, xlsx o csv) fueron descargadas y limpiadas en el programa STATA 18.0 para Windows. Luego se procedió a unir la base de casos y muertes por la COVID-19, mediante el identificador único y las fechas de infección y defunción. De esta forma se incorporaron como nuevos registros a las defunciones que no estaban contemplados como casos previamente, obteniendo una base con los siguientes datos para cada registro: identificador único, sexo, grupo de edad, fecha de caso y ubigeo del distrito de residencia; añadiéndose una columna de “caso fallecido”, marcándose como “sí”, cuando durante el periodo de infección (±90 días) se registró la defunción. Tras ello, según el ubigeo de residencia, se asignó la altitud distrital, el IDH y la densidad poblacional, agregándose dichos datos como columnas adicionales a la base antes mencionada y para cada registro individualmente. Posteriormente, se agregó el estado de vacunación y la hospitalización según el identificador único y la fecha de caso, como dos columnas adicionales, de forma que, si el caso contaba con al menos una inmunización a la fecha de caso, esto se reportó como "sí vacunado", incluyendo las dosis recibidas hasta dicho momento; y si reportaba una hospitalización por la COVID-19 en el periodo de ±90 días desde la fecha de caso, se reportó como “sí hospitalizado”. Finalmente, según la fecha de caso, se adicionaron los datos correspondientes a la disponibilidad de recursos sanitarios por distrito de residencia, agregándose el dato de cada registro como una columna adicional, y se estableció la categoría de ola epidémica, agregando este dato como una última columna adicional.

Los datos de las bases a utilizar tuvieron como período de tiempo de marzo del 2020 hasta diciembre del 2022, y se obtuvieron de los siguientes lugares tomando las siguientes consideraciones:

Casos de COVID-19: se empleó la base de datos del MINSA (base de datos dinámica) para los casos reportados en el territorio peruano, tomando en cuenta el periodo de ± 90 días para considerar como un nuevo episodio de COVID-19 al reporte de una misma persona en esta base de datos, unificando los registros que se encontraban comprendidos en este mismo periodo de tiempo (considerando únicamente al primer reporte como el indicador de fecha en la que se consideró como caso a la persona).

https://www.datosabiertos.gob.pe/dataset/casos-positivos-por-covid-19-ministerio-de-salud-minsa.

Muertes por COVID-19: también se empleó la base de datos del MINSA (base de datos dinámica que incluía las muertes según los 7 criterios anteriormente expuestos), la cual se superpuso a la base de casos de COVID-19 según el identificador único. En los casos de las defunciones que no estaban reportadas como casos previamente, estas fueron incorporadas como casos de COVID-19 incluyendo a la fecha de defunción como la fecha en la que se consideró como caso a la persona (esto ocurrió, por ejemplo, para defunciones por criterios de investigación y nexo epidemiológico, donde no se contó con pruebas positivas que determinaran el inicio de la enfermedad, pero se determinó a la defunción como producto de dicha enfermedad).

https://www.datosabiertos.gob.pe/dataset/fallecidos-por-covid-19-ministerio-de-salud-minsa.

Altitud según distrito: se tomó en cuenta la altitud distrital de residencia según el distrito reportado (obtenido a partir de los códigos de UBIGEO reportados para cada distrito) en la base de casos, en primer lugar, pasando a ser complementado por el distrito de residencia de la base de defunciones, en caso el dato no se hubiera obtenido en la primera base mencionada. El distrito de residencia era reportado según el Registro Nacional de Identificación y Estado Civil (RENIEC), el cual fue vinculado a la altitud del distrito reportada en la base del MINSA, que obtenía la información del INEI.

https://www.datosabiertos.gob.pe/dataset/codigos-equivalentes-de-ubigeo-del-peru.

Vacunación contra la COVID-19: se utilizó la base de datos del MINSA (base de datos dinámica), la cual incluía el reporte de todas las inmunizaciones contra la COVID-19 de forma individual y según el identificador único de cada persona, incluyendo la fecha de administración de cada dosis (lo que permitió conocer el estado de vacunación al compararlo con la fecha de caso).

https://www.datosabiertos.gob.pe/dataset/vacunacion.

Hospitalizaciones por COVID-19: se usó la base de datos del MINSA (base de datos dinámica), la cual incluía el reporte de hospitalizaciones en el territorio nacional según el identificador único, incluyendo las fechas de internamiento (lo que permitió conocer si el caso fue hospitalizado durante el episodio de COVID-19, según la fecha de caso).

https://www.datosabiertos.gob.pe/dataset/hospitalizados-vacunados-y-fallecidos-por-covid-19.

Densidad poblacional: en base al distrito de procedencia, se estimó la densidad poblacional a partir de la población total estimada por distrito por el INEI para el año 2020, dato que se dividió entre superficie distrital (en km^2^) para obtener el valor final incluido en la base de datos.

https://www.minsa.gob.pe/reunis/data/poblacion_estimada.asp.

El dato de la superficie distrital se obtuvo de la misma base de datos que contenía las altitudes distritales, la cual fue mencionada anteriormente, siendo estos datos obtenidos de los reportes del INEI.

https://www.datosabiertos.gob.pe/dataset/codigos-equivalentes-de-ubigeo-del-peru.

Índice de Desarrollo Humano: este dato fue atribuido tomando en cuenta el reporte del Instituto Peruano de Economía 2019 para cada distrito del territorio nacional.

https://www.ipe.org.pe/portal/indice-de-desarrollo-humano-idh/.

Camas de hospitalización, de UCI, de UCIN y camillas de emergencia disponibles: estos datos fueron obtenidos tomando en cuenta el registro de SUSALUD del Perú (base de datos dinámica), quienes brindaron un registro de las camas de hospitalización, de UCI, de UCIN y de las camillas de emergencia disponibles por cada establecimiento de salud, indicando el distrito al que pertenecía cada establecimiento. De esta forma se pudo llevar un registro de recursos de salud disponibles para recibir pacientes por distrito. Este registro se realizó diariamente, pero lamentablemente no se contó con un registro completo para todo el periodo de tiempo de estudio (el reporte fue inconsistente en los diferentes establecimientos de salud a lo largo de la pandemia de COVID-19, y no hubo reporte alguno para los periodos comprendido durante la primera ni la quinta ola epidémica en el Perú).

http://datos.susalud.gob.pe/dataset/data-hist%C3%B3rica-del-registro-de-camas-diarias-disponibles-y-ocupadas-del-formato-f5002-v2-2#{}.

Los datos provienen de los registros oficiales del MINSA, los cuales dependen de los sistemas de vigilancia epidemiológica regionales. Dado que el grado de notificación puede variar entre jurisdicciones, especialmente en distritos remotos o de mayor altitud, reconocemos la posibilidad de diferencias en la completitud del registro de casos y defunciones. Esta potencial variabilidad en la captación fue considerada en la interpretación de los resultados y se discute como posible fuente de sesgo de selección.

### Análisis estadístico

Primero se realizó un análisis descriptivo, reportando frecuencias y porcentajes. También se hizo un análisis descriptivo específico según olas epidémicas y categorías de altitud.

Luego se realizó un análisis bivariado para evaluar la relación de la muerte por la COVID-19 con la altitud, así como se evaluó la relación de la muerte por la COVID-19 con el sexo, grupo de edad, vacunación, hospitalización, densidad poblacional e IDH, empleando la prueba de Chi^2^. Esto se realizó globalmente y por olas epidémicas.

Se empleó una regresión de Poisson con varianza robusta agrupada por distrito, tomando como desenlace a la muerte por la COVID-19, estimando el riesgo relativo (RR) con intervalos de confianza al 95% (IC95%). Las covariables para ajustar el modelo multivariable fueron: sexo, grupo de edad, vacunación, hospitalización, IDH y densidad poblacional. Se procedió a realizar el análisis global, así como por olas epidémicas. Para el análisis global se ajustó además por ola epidémica, y en el caso de la regresión múltiple de la primera ola epidémica no se empleó a la vacunación, debido a que para entonces aún no se había iniciado la vacunación a nivel nacional. Se tomó en cuenta el supuesto de dependencia intra-cluster, reconociendo que las observaciones dentro de un mismo distrito podrían estar correlacionadas, pero asumiendo independencia entre distritos, y se evaluó la multicolinealidad mediante el factor de inflación de la varianza (VIF), a través de un modelo lineal auxiliar con los mismos predictores del modelo principal.

Dado que hubo una gran cantidad de datos ausentes para el registro de recursos sanitarios disponibles, se optó por excluirlos del análisis principal. No obstante, se realizó un análisis de sensibilidad incluyendo a la disponibilidad de recursos de salud en las regresiones (a nivel global y por olas epidémicas). Asimismo, se realizó un análisis de sensibilidad para evaluar únicamente a las muertes de los casos con pruebas positivas previas. Así, se repitieron las regresiones eliminando a las muertes que no estaban registradas como casos inicialmente, tanto a nivel global como por olas epidémicas. Por último, también se realizó un análisis de sensibilidad adicional evaluando la relación de la altitud y la muerte por COVID-19 tomando en cuenta la clasificación de la altitud según la clasificación alternativa más alineada con los pisos altitudinales de Pulgar Vidal, repitiendo las regresiones tanto a nivel global como por olas epidémicas.

Se consideró al valor de p<0,05 como estadísticamente significativo para todos los análisis realizados. Para realizar todo el plan de análisis descrito se utilizó el programa STATA 18.0 para Windows (Stata Corp., College Station, TX).

### Aspectos éticos

El estudio utilizó bases secundarias de acceso abierto. La variable de identificación provino de un código encriptado, por lo que no existió la posibilidad de identificar a la persona incluida en los registros. Este protocolo fue registrado en el Sistema Descentralizado de Información y Seguimiento a la Investigación (SIDISI) - Dirección Universitaria de Investigación, Ciencia y Tecnología (DUICT), bajo el código 213552, y fue evaluado por el Comité de Ética de la UPCH (CIE-UPCH) previo a su ejecución, siendo aprobado bajo el código CIEI-109-10-24. Asimismo, se realizó el registro en la plataforma de Proyectos de Investigación en Salud (PRISA) bajo el código EI00003486. Durante el desarrollo del estudio se respetaron los principios éticos delineados en la Declaración de Helsinki, y se siguieron estrictamente las recomendaciones brindadas por el CIE-UPCH.

## RESULTADOS

Se obtuvo un total de 4 331 784 casos de la COVID-19 en Perú entre marzo de 2020 y diciembre de 2022, pero se excluyeron 211 015 registros debido a que no contaron con el identificador único (2,0%) y/o distrito de residencia (2,9%), obteniéndose un total de 4 120 769 casos analizables.

Del total analizable, 51,6% fueron mujeres, el 68,9% tenía menos de 50 años y el 53,0% no se había inmunizado cuando tuvo la COVID-19. La mayor proporción residía en un distrito con IDH medio (45,2%) y con alta densidad poblacional (66,5%). Asimismo, la mayoría residía entre 0 y 500 msnm (69,9%). Finalmente, se registraron 215 423 muertes (5,2%). Esta información, a nivel global como por olas epidémicas, se encuentra en la tabla 1. Asimismo, se presentan las características descritas para cada categoría de altitud en la material suplementario 1.

**Tabla 1:** Características de los pacientes con COVID-19 global y según ola epidémica, Perú 2020-2022.

Características		Total	Ola epidémica				
			1ra ola	2da ola	3ra ola	4ta ola	5ta ola
		n=4 120 769 (%)	n=952 811(%)	n= 1 280 781(%)	n=1 171 485 (%)	n=567 318(%)	n=148 374(%)
Sexo							
	Femenino	2 124 796 (51,6)	458 153 (48,1)	633 048 (49,4)	627 615 (53,6)	321 576 (56,7)	84 404 (56,9)
	Masculino	1 995 973 (48,4)	494 658 (51,9)	647 733 (50,6)	543 870 (46,4)	245 742 (43,3)	63 970 (43,1)
Grupo de edad*							
	0 a 49 años	2 838 887 (68,9)	609 792 (64,0)	852 074 (66,5)	898 626 (76,7)	387 433 (68,3)	90 962 (61,3)
	50 a 74 años	1 078 263 (26,2)	283 661 (29,8)	356 519 (27,8)	235 082 (20,1)	154 889 (27,3)	48 112 (32,4)
	75 años a más	203 469 (4,9)	59 336 (6,2)	72 063 (5,6)	37 775 (3,2)	24 995 (4,4)	9 300 (6,3)
Vacunación							
	No	2 183 238 (53,0)	952 809 (∼100,0)	1 110 002 (86,7)	99 824 (8,5)	17 590 (3,1)	3 013 (2,0)
	1 dosis	110 853 (2,7)	0 (0,0)	48 765 (3,8)	46 260 (4,0)	13 693 (2,4)	2 135 (1,4)
	2 dosis	815 739 (19,8)	2 (∼0,0)	115 313 (9,0)	636 415 (54,3)	55 559 (9,8)	8 450 (5,7)
	3 o más dosis	1 010 939 (24,5)	0 (0,0)	6 701 (0,5)	388 986 (33,2)	480 476 (84,7)	134 776 (90,8)
Hospitalización							
	No	4 038 276 (98,0)	922 674 (96,8)	1 235 012 (96,4)	1 166 472 (99,6)	566 047 (99,8)	148 071 (99,8)
	Sí	82 493 (2,0)	30 137 (3,2)	45 769 (3,6)	5 013 (0,4)	1 271 (0,2)	303 (0,2)
Índice de desarrollo humano							
	Bajo	600 005 (14,6)	160 514 (16,8)	225 903 (17,6)	143 983 (12,3)	55 831 (9,8)	13 774 (9,3)
	Medio	1 861 734 (45,2)	471 486 (49,5)	571 442 (44,6)	524 852 (44,8)	226 670 (40,0)	67 284 (45,4)
	Alto	1 146 023 (27,8)	252 973 (26,6)	344 652 (26,9)	333 414 (28,5)	172 042 (30,3)	42 942 (28,9)
	Muy alto	513 007 (12,4)	67 838 (7,1)	138 784 (10,8)	169 236 (14,4)	112 775 (19,9)	24 374 (16,4)
Densidad poblacional							
	Baja	718 106 (17,4)	193 364 (20,3)	249 515 (19,5)	181 643 (15,5)	73 228 (12,9)	20 356 (13,7)
	Media	661 123 (16,0)	165 586 (17,4)	213 710 (16,7)	184 626 (15,8)	74 592 (13,2)	22 609 (15,2)
	Alta	2 741 540 (66,5)	593 861 (62,3)	817 556 (63,8)	805 216 (68,7)	419 498 (73,9)	105 409 (71,0)
Altitud distrital de residencia							
	0 a 500 msnm	2 878 797 (69,9)	693 839 (72,8)	859 704 (67,1)	818 372 (69,9)	401 296 (70,7)	105 586 (71,2)
	501 a 2000 msnm	350 773 (8,5)	88 908 (9,3)	118 107 (9,2)	92 316 (7,9)	40 093 (7,1)	11 349 (7,6)
	2001 a 3500 msnm	745 509 (18,1)	136 273 (14,3)	252 788 (19,7)	219 335 (18,7)	110 162 (19,4)	26 951 (18,2)
	3501 msnm a más	145 690 (3,5)	33 791 (3,6)	50 182 (3,9)	41 462 (3,5)	15 767 (2,8)	4 488 (3,0)
Muerte por COVID-19							
	No	3 905 346 (94,8)	859 572 (90,2)	1 171 565 (91,5)	1 161 665 (99,2)	564 630 (99,5)	147 914 (99,7)
	Sí	215 423 (5,2)	93 239 (9,8)	109 216 (8,5)	9 820 (0,8)	2 688 (0,5)	460 (0,3)

* Para esta variable el 100% puede ser menor que el global por falta de registros (se contó con 150 registros ausentes).

En el análisis bivariado, se encontró una asociación estadísticamente significativa (p<0,001) entre la muerte por la COVID-19 y la altitud, evidenciando que las personas que residían entre 0 y 500 msnm, así como las que residían de 3501 msnm a más, fueron las que más muertes reportaron (con 5,7% y 5,6%, respectivamente). También se encontró una asociación estadísticamente significativa (p<0,001) con las variables de sexo, grupo de edad, vacunación, hospitalización, IDH y densidad poblacional. Estos resultados se presentan en la tabla 2. Todas las asociaciones presentadas también fueron evaluadas por olas epidémicas, lo que se puede visualizar en la tabla 3.

**Tabla 2:** Características asociadas a la muerte por COVID-19, Perú 2020-2022, análisis bivariado global.

Características		Muerte por COVID-19		p
		No (n=3 905 346)	Sí (n=215 423)	
		n (%)	n (%)	
Altitud distrital de residencia				<0,001
	0 a 500 msnm	2 715 947 (94,3)	162 850 (5,7)	
	501 a 2000 msnm	336 325 (95,9)	14 448 (4,1)	
	2001 a 3500 msnm	715 580 (96,0)	29 929 (4,0)	
	3501 msnm a más	137 494 (94,4)	8 196 (5,6)	
Sexo				<0,001
	Femenino	2 047 024 (96,3)	77 772 (3,7)	
	Masculino	1 858 322 (93,1)	137 651 (6,9)	
Grupos de edad*				<0,001
	0 a 49 años	2 809 108 (99,0)	29 779 (1,0)	
	50 a 74 años	962 769 (89,3)	115 494 (10,7)	
	75 años a más	133 319 (65,5)	70 150 (34,5)	
Vacunación				<0,001
	No	1 985 976 (91,0)	197 262 (9,0)	
	1 dosis	105 665 (95,3)	5 188 (4,7)	
	2 dosis	807 569 (99,0)	8 170 (1,0)	
	3 o más dosis	1 006 136 (99,5)	4 803 (0,5)	
Hospitalización				<0,001
	No	3 860 065 (95,6)	178 211 (4,4)	
	Sí	45 281 (54,9)	37 212 (45,1)	
Índice de desarrollo humano				<0,001
	Bajo	565 693 (94,3)	34 312 (5,7)	
	Medio	1 755 012 (94,3)	106 722 (5,7)	
	Alto	1 086 538 (94,8)	59 485 (5,2)	
	Muy alto	498 103 (97,1)	14 904 (2,9)	
Densidad poblacional				<0,001
	Baja	679 439 (94,6)	38 667 (5,4)	
	Media	621 740 (94,0)	39 383 (6,0)	
	Alta	2 604 167 (95,0)	137 373 (5,0)	

* Para esta variable el 100% puede ser menor que el global por falta de registros (se contó con 150 registros ausentes).

**Tabla 3:** Características asociadas a la muerte por COVID-19, Perú 2020-2022, análisis bivariado estratificado por ola epidémica.

Características		Muerte por COVID-19 (1ra ola)		p	Muerte por COVID-19 (2da ola)		p	Muerte por COVID-19 (3ra ola)		p	Muerte por COVID-19 (4ta ola)		p	Muerte por COVID-19 (5ta ola)		p
		No (n=859 572)	Sí (n=93 239)		No (n=1 171 565)	Sí (n=109 216)		No (n=1 161 665)	Sí (n=9 820)		No (n=564 630)	Sí (n=2 688)		No (n=147 914)	Sí (n=460)	
		n (%)	n (%)		n (%)	n (%)		n (%)	n (%)		n (%)	n (%)		n (%)	n (%)	
Altitud distrital de residencia				<0,001			<0,001			<0,001			<0,001			0,009
	0 a 500 msnm	619 279 (89,3)	74 560 (10,7)		780 333 (90,8)	79 371 (9,2)		811 529 (99,2)	6 843 (0,8)		399 574 (99,6)	1 722 (0,4)		105 232 (99,7)	354 (0,3)	
	501 a 2000 msnm	83 308 (93,7)	5 600 (6,3)		110 302 (93,4)	7 805 (6,6)		91 568 (99,2)	748 (0,8)		39 836 (99,4)	257 (0,6)		11 311 (99,7)	38 (0,3)	
	2001 a 3500 msnm	125 632 (92,2)	10 641 (7,8)		235 666 (93,2)	17 122 (6,8)		217 770 (99,3)	1 565 (0,7)		109 618 (99,5)	544 (0,5)		26 894 (99,8)	57 (0,2)	
	3501 msnm a más	31 353 (92,8)	2 438 (7,2)		45 264 (90,2)	4 918 (9,8)		40 798 (98,4)	664 (1,6)		15 602 (99,0)	165 (1,0)		4 477 (99,8)	11 (0,2)	
Sexo				<0,001			<0,001			<0,001			<0,001			<0,001
	Femenino	427 038 (93,2)	31 115 (6,8)		592 023 (93,5)	41 025 (6,5)		623 348 (99,3)	4 267 (0,7)		320 412 (99,6)	1 164 (0,4)		84 203 (99,8)	201 (0,2)	
	Masculino	432 534 (87,4)	62 124 (12,6)		579 542 (89,5)	68 191 (10,5)		538 317 (99,0)	5 553 (1,0)		244 218 (99,4)	1 524 (0,6)		63 711 (99,6)	259 (0,4)	
Grupo de edad*				<0,001			<0,001			<0,001			<0,001			<0,001
	0 a 49 años	597 800 (98,0)	11 992 (2,0)		836 561 (98,2)	15 513 (1,8)		896 770 (99,8)	1 856 (0,2)		387 054 (99,9)	379 (0,1)		90 923 (∼100,0)	39 (∼0,0)	
	50 a 74 años	232 369 (81,9)	51 292 (18,1)		296 48 (83,2)	60 091 (16,9)		231 897 (98,6)	3 185 (1,4)		154 096 (99,5)	793 (0,5)		47 979 (99,7)	133 (0,3)	
	75 años a más	29 381 (49,5)	29 955 (50,5)		38 451 (53,4)	33 612 (46,6)		32 996 (87,3)	4 779 (12,7)		23 479 (93,9)	1 516 (6,1)		9 012 (96,9)	288 (3,1)	
Vacunación							<0,001			<0,001			<0,001			<0,001
	No	-	-	-	1 009 400 (90,9)	100 602 (9,1)		96 769 (96,9)	3 055 (3,1)		17 263 (98,1)	327 (1,9)		2 974 (98,7)	39 (1,3)	
	1 dosis	-	-	-	44 180 (90,6)	4 585 (9,4)		45 736 (98,9)	524 (1,1)		13 622 (99,5)	71 (0,5)		2 127 (99,6)	8 (0,4)	
	2 dosis	-	-	-	111 399 (96,6)	3 914 (3,4)		632 648 (99,4)	3 767 (0,6)		55 122 (99,2)	437 (0,8)		8 398 (99,4)	52 (0,6)	
	3 o más dosis	-	-	-	6 586 (98,3)	115 (1,7)		386 512 (99,4)	2 474 (0,6)		478 623 (99,6)	1 853 (0,4)		134 415 (99,7)	361 (0,3)	
Hospitalización				<0,001			<0,001			<0,001			<0,001			<0,001
	No	843 861 (91,5)	78 813 (8,5)		1 146 907 (92,9)	88 105 (7,1)		1 158 066 (99,3)	8 406 (0,7)		563 593 (99,6)	2 454 (0,4)		147 638 (99,7)	433 (0,3)	
	Sí	15 711 (52,1)	14 426 (47,9)		24 658 (53,9)	21 111 (46,1)		3 599 (71,8)	1 414 (28,2)		1 037 (81,6)	234 (18,4)		276 (91,1)	27 (8,9)	
Índice de desarrollo humano				<0,001			<0,001			<0,001			<0,001			0,022
	Bajo	147 654 (92,0)	1 860 (8,0)		207 078 (91,7)	18 825 (8,3)		141 935 (98,6)	2 048 (1,4)		55 313 (99,1)	518 (0,9)		13 713 (99,6)	61 (0,4)	
	Medio	423 498 (89,8)	47 988 (10,2)		518 586 (90,8)	52 856 (9,2)		520 405 (99,2)	4 447 (0,8)		225 446 (99,5)	1 224 (0,5)		67 077 (99,7)	207 (0,3)	
	Alto	225 991 (89,3)	26 982 (10,7)		315 275 (91,5)	29 377 (8,5)		331 031 (99,3)	2 383 (0,7)		171 426 (99,6)	616 (0,4)		42 815 (99,7)	127 (0,3)	
	Muy alto	62 429 (92,0)	5 409 (8,0)		130 626 (94,1)	8 158 (5,9)		168 294 (99,4)	942 (0,6)		112 445 (99,7)	330 (0,3)		24 309 (99,7)	65 (0,3)	
Densidad poblacional				<0,001			<0,001			<0,001			<0,001			0,009
	Baja	178 715 (92,4)	14 649 (7,6)		228 496 (91,6)	21 019 (8,4)		179 339 (98,7)	2 304 (1,3)		72 610 (99,2)	618 (0,8)		20 279 (99,6)	77 (0,4)	
	Media	148 489 (89,7)	17 097 (10,3)		193 746 (90,7)	19 964 (9,3)		182 887 (99,1)	1 739 (0,9)		74 095 (99,3)	497 (0,7)		22 523 (99,6)	89 (0,4)	
	Alta	532 368 (89,6)	61 493 (10,4)		749 323 (91,7)	68 233 (8,3)		799 439 (99,3)	5 777 (0,7)		417 925 (99,6)	1 573 (0,4)		105 112 (99,7)	460 (0,3)	

* Para esta variable el 100% puede ser menor que el global por falta de registros (se contó con 150 registros ausentes).

En el análisis multivariable para evaluar la relación de la muerte por la COVID-19 y la altitud (ajustado por sexo, grupo de edad, vacunación, hospitalización, IDH, densidad poblacional y ola epidémica) se evidenció una asociación estadísticamente significativa. Comparado con la altitud de 0-500 msnm, los residentes de 501-2000 msnm tenían 32% (IC95%: 31% a 34%) menor riesgo de muerte y los residentes de 2001-3500 msnm tenían 31% (IC95%: 30% a 32%) menor riesgo de muerte, mientras que los residentes de ≥3501 msnm no tenían diferencias en cuanto al riesgo de muerte (p=0,498). Estos resultados pueden visualizarse en la tabla 4. Adicionalmente, en la tabla 5 se puede observar el análisis realizado por olas epidémicas, en donde se puede evidenciar una tendencia similar en los modelos multivariable ajustados, obteniendo consistentemente un menor riesgo de muerte en la categoría de 2001-3500 msnm en comparación con el nivel del mar en todas las olas epidémicas.

**Tabla 4:** Asociación entre altitud distrital de residencia y la muerte por COVID-19, Perú 2020-2022, enfoque epidemiológico a nivel global.

Características		Análisis bivariado		p	Regresión múltiple*^✝^		p
		RR	(IC 95%)		RR	(IC 95%)	
Altitud distrital de residencia							
	0 a 500 msnm	Ref.		-	Ref.		-
	501 a 2000 msnm	0,73	(0,62-0,85)	<0,001	0,67	(0,61-0,74)	<0,001
	2001 a 3500 msnm	0,71	(0,63-0,80)	<0,001	0,68	(0,63-0,74)	<0,001
	3501 msnm a más	0,99	(0,88-1,13)	0,932	0,98	(0,88-1,09)	0,666
Sexo							
	Femenino	Ref.		-	Ref.		-
	Masculino	1,88	(1,85-1,91)	<0,001	1,52	(1,51-1,54)	<0,001
Grupo de edad^✝^							
	0 a 49 años	Ref.		-	Ref.		-
	50 a 74 años	10,21	(9,93-10,50)	<0,001	8,46	(8,21-8,71)	<0,001
	75 años a más	32,87	(32,43-34,38)	<0,001	24,45	(23,24-25,72)	<0,001
Vacunación							
	No	Ref.		-	Ref.		-
	1 dosis	0,52	(0,46-0,58)	<0,001	0,71	(0,68-0,74)	<0,001
	2 dosis	0,11	(0,10-0,12)	<0,001	0,35	(0,33-0,37)	<0,001
	3 o más dosis	0,05	(0,05-0,06)	<0,001	0,15	(0,14-0,16)	<0,001
Hospitalización							
	No	Ref.		-	Ref.		-
	Sí	10,22	(9,61-10,88)	<0,001	3,29	(3,16-3,43)	<0,001
Índice de desarrollo humano							
	Bajo	Ref.		-	Ref.		-
	Medio	1,00	(0,91-1,11)	0,962	1,02	(0,94-1,11)	0,590
	Alto	0,91	(0,78-1,05)	0,198	0,90	(0,81-1,02)	0,091
	Muy alto	0,51	(0,40-0,64)	<0,001	0,67	(0,55-0,81)	<0,001
Densidad poblacional							
	Baja	Ref.		-	Ref.		-
	Media	1,11	(0,97-1,26)	0,139	1,04	(0,94-1,15)	0,455
	Alta	0,93	(0,81-1,07)	0.301	1,13	(1,03-1,23)	0,007
Ola epidémica							
	1ra ola	Ref.		-	Ref.		-
	2da ola	0,87	(0,83-0,91)	<0,001	1,03	(0,99-1,07)	0.111
	3ra ola	0,09	(0,08-0,09)	<0,001	0,48	(0,44-0,52)	<0,001
	4ta ola	0,05	(0,04-0,05)	<0,001	0,36	(0,32-0,40)	<0,001
	5ta ola	0,03	(0,03-0,04)	<0,001	0,21	(0,18-0,24)	<0,001

* Modelo de regresión de Poisson con varianzas robustas agrupadas por distrito, ajustado por variables individuales (sexo, grupo de edad, vacunación, hospitalización) y contextuales (índice de desarrollo humano, densidad poblacional, ola epidémica).

✝ Para esta variable y modelo se contó con 4 120 619 registro.

RR: Riesgo relativo; IC 95%: Intervalo de confianza al 95%.

**Tabla 5:** Asociación entre altitud distrital de residencia y la muerte por COVID-19, Perú 2020-2022, enfoque epidemiológico según ola epidémica.

Características		1ra ola (n=952 811)						2da ola (n= 1 280 781)						3ra ola (n=1 171 485)						4ta ola (n=567 318)						5ta ola (n=148 374)					
		Análisis bivariado		p	Regresión múltiple*✝		p	Análisis bivariado		p	Regresión múltiple**✝✝		p	Análisis bivariado		p	Regresión múltiple**✝✝✝		p	Análisis bivariado		p	Regresión múltiple**✝✝✝✝		p	Análisis bivariado		p	Regresión múltiple**		p
		RR	(IC 95%)		RR	(IC 95%)		RR	(IC 95%)		RR	(IC 95%)		RR	(IC 95%)		RR	(IC 95%)		RR	(IC 95%)		RR	(IC 95%)		RR	(IC 95%)		RR	(IC 95%)	
Altitud distrital de residencia																															
	0 a 500 msnm	Ref.		-	Ref.		-	Ref.		-	Ref.		-	Ref.		-	Ref.		-	Ref.		-	Ref.		-	Ref.		-	Ref.		-
	501 a 2000 msnm	0,59	(0,52-0,67)	<0,001	0,65	(0,58-0,73)	<0,001	0,72	(0,61-0,84)	<0,001	0,68	(0,61-0,77)	<0,001	0,97	(0,80-1,17)	0,749	0,62	(0,53-0,72)	<0,001	1,49	(1,17-1,90)	0,001	0,86	(0,69-1,06)	0,156	1,00	(0,65-1,54)	0,995	0,76	(0,48-1,19)	0,233
	2001 a 3500 msnm	0,73	(0,65-0,81)	<0,001	0,72	(0,65-0,79)	<0,001	0,73	(0,65-0,82)	<0,001	0,66	(0,60-0,73)	<0,001	0,85	(0,71-1,03)	0,096	0,67	(0,60-0,74)	<0,001	1,15	(0,92-1,43)	0,210	0,67	(0,57-0,79)	<0,001	0,63	(0,47-0,85)	0,003	0,45	(0,34-0,60)	<0,001
	3501 msnm a más	0,67	(0,59-0,76)	<0,001	0,87	(0,79-0,97)	0,014	1,06	(0,95-1,18)	0,286	1,00	(0,87-1,14)	0,989	1,92	(1,50-2,44)	<0,001	1,01	(0,83-1,23)	0,933	2,44	(1,77-3,36)	<0,001	1,07	(0,77-1,48)	0,697	0,73	(0,43-1,25)	0,253	0,50	(0,29-0,88)	0,015
Sexo																															
	Femenino	Ref.		-	Ref.		-	Ref.		-	Ref.		-	Ref.		-	Ref.		-	Ref.		-	Ref.		-	Ref.		-	Ref.		-
	Masculino	1,85	(1,81-1,89)	<0,001	1,59	(1,56-1,61)	<0,001	1,62	(1,59-1,66)	<0,001	1,48	(1,46-1,51)	<0,001	1,50	(1,43-1,58)	<0,001	1,42	(1,37-1,48)	<0,001	1,71	(1,59-1,85)	<0,001	1,59	(1,47-1,71)	<0,001	1,70	(1,45-2,00)	<0,001	1,57	(1,34-1,86)	<0,001
Grupo de edad																															
	0 a 49 años	Ref.		-	Ref.		-	Ref.		-	Ref.		-	Ref.		-	Ref.		-	Ref.		-	Ref.		-	Ref.		-	Ref.		-
	50 a 74 años	9,19	(8,92-9,48)	<0,001	8,29	(8,03-8,57)	<0,001	9,26	(8,94-9,58)	<0,001	8,28	(7,97-8,60)	<0,001	6,56	(6,14-7,01)	<0,001	8,57	(8,04-9,14)	<0,001	5,23	(4,56-6,00)	<0,001	6,43	(5,58-7,41)	<0,001	6,45	(4,50-9,23)	<0,001	7,17	(5,00-10,30)	<0,001
	75 años a más	25,67	(25,58-26,81)	<0,001	22,14	(21,16-23,16)	<0,001	25,62	(24,24-27,08)	<0,001	22,50	(21,20-23,77)	<0,001	61,25	(56,13-66,84)	<0,001	64,60	(58,26-71,64)	<0,001	62,00	(53,79-71,47)	<0,001	62,73	(54,28-72,49)	<0,001	72,23	(50,69-102,93)	<0,001	72,27	(50,32-103,80)	<0,001
Vacunación																															
	No	-		-	-		-	Ref.		-	Ref.		-	Ref.		-	Ref.		-	Ref.		-	Ref.		-	Ref.		-	Ref.		-
	1 dosis	-		-	-		-	1,04	(0,98-1,10)	0,224	0,78	(0,75-0,81)	<0,001	0,37	(0,32-0,43)	<0,001	0,44	(0,39-0,49)	<0,001	0,28	(0,21-0,37)	<0,001	0,47	(0,37-0,60)	<0,001	0,29	(0,14-0,61)	0,001	0,49	(0,24-0,98)	0,044
	2 dosis	-		-	-		-	0,37	(0,35-0,40)	<0,001	0,39	(0,37-0,41)	<0,001	0,19	(0,17-0,21)	<0,001	0,30	(0,27-0,32)	<0,001	0,42	(0,36-0,50)	<0,001	0,59	(0,51-0,68)	<0,001	0,48	(0,31-0,72)	<0,001	0,65	(0,43-1,00)	0,048
	3 o más dosis	-		-	-		-	0,19	(0,16-0,23)	<0,001	0,14	(0,11-0,16)	<0,001	0,21	(0,19-0,23)	<0,001	0,13	(0,12-0,14)	<0,001	0,21	(0,18-0,24)	<0,001	0,22	(0,19-0,25)	<0,001	0,21	(0,15-0,29)	<0,001	0,24	(0,17-0,34)	<0,001
Hospitalización																															
	No	Ref.		-	Ref.		-	Ref.		-	Ref.		-	Ref.		-	Ref.		-	Ref.		-	Ref.		-	Ref.		-	Ref.		-
	Sí	5,60	(5,26-5,97)	<0,001	3,09	(2,96-3,23)	<0,001	6,47	(6,04-6,92)	<0,001	3,32	(3,15-3,49)	<0,001	39,14	(35,08-43,67)	<0,001	5,35	(4,87-5,89)	<0,001	42,47	(35,11-51,37)	<0,001	6,53	(5,47-7,79)	<0,001	30,47	(19,78-46,95)	<0,001	7,04	(4,19-11,84)	<0,001
Índice de desarrollo humano																															
	Bajo	Ref.		-	Ref.		-	Ref.		-	Ref.		-	Ref.		-	Ref.		-	Ref.		-	Ref.		-	Ref.		-	Ref.		-
	Medio	1,27	(1,12-1,44)	<0,001	1,00	(0,91-1,11)	0,989	1,11	(1,01-1,22)	0,032	1,08	(0,97-1,19)	0,158	0,60	(0,53-0,67)	<0,001	0,87	(0,78-0,98)	0,019	0,58	(0,50-0,68)	<0,001	0,82	(0,68-0,98)	0,033	0,69	(0,51-0,94)	0,019	0,66	(0,48-0,90)	0,009
	Alto	1,33	(1,17-1,51)	<0,001	0,92	(0,81-1,05)	0,204	1,02	(0,88-1,19)	0,766	0,93	(0,80-1,07)	0,324	0,50	(0,41-0,61)	<0,001	0,74	(0,60-0,92)	0,006	0,39	(0,32-0,47)	<0,001	0,59	(0,45-0,76)	<0,001	0,67	(0,48-0,93)	0,018	0,58	(0,39-0,86)	0,006
	Muy alto	1,00	(0,76-1,30)	0,972	0,68	(0,56-0,83)	<0,001	0,71	(0,56-0,89)	0,004	0,70	(0,57-0,88)	0,002	0,39	(0,30-0,51)	<0,001	0,52	(0,40-0,69)	<0,001	0,32	(0,25-0,40)	<0,001	0,41	(0,30-0,55)	<0,001	0,60	(0,39-0,94)	0,024	0,41	(0,25-0,66)	<0,001
Densidad poblacional																															
	Baja	Ref.		-	Ref.		-	Ref.		-	Ref.		-	Ref.		-	Ref.		-	Ref.		-	Ref.		-	Ref.		-	Ref.		-
	Media	1,36	(1,18-1,58)	<0,001	1,12	(0,99-1,26)	0,063	1,11	(0,98-1,26)	0,109	1,00	(0,89-1,12)	0,999	0,74	(0,62-0,89)	0,002	0,89	(0,78-1,00)	0,057	0,79	(0,65-0,96)	0,020	0,95	(0,78-1,16)	0,603	1,01	(0,72-1,41)	0,974	1,09	(0,75-1,58)	0,664
	Alta	1,37	(1,22-1,53)	<0,001	1,20	(1,08-1,34)	0,001	0,99	(0,88-1,12)	0,883	1,10	(0,98-1,23)	0,105	0,57	(0,48-0,67)	<0,001	0,95	(0,84-1,08)	0,474	0,44	(0,37-0,53)	<0,001	0,78	(0,64-0,95)	0,011	0,74	(0,56-1,00)	0,047	0,95	(0,65-1,39)	0,794

* Modelo de regresión de Poisson con varianzas robustas agrupadas por distrito, ajustado por variables individuales (sexo, grupo de edad, hospitalización) y contextuales (índice de desarrollo humano, densidad poblacional).

** Modelo de regresión de Poisson con varianzas robustas agrupadas por distrito, ajustado por variables individuales (sexo, grupo de edad, vacunación, hospitalización) y contextuales (índice de desarrollo humano, densidad poblacional).

✝ Para este modelo se contó con 952 789 registros.

✝✝ Para este modelo se contó con 1 280 656 registros.

✝✝✝ Para este modelo se contó con 1 171 483 registros.

✝✝✝✝ Para este modelo se contó con 567 317 registros.

RR: Riesgo relativo; IC 95%: Intervalo de confianza al 95%.

Finalmente, respecto a los análisis de sensibilidad, tanto para la de disponibilidad de recursos de salud a nivel global (material suplementario 2) como por olas epidémicas (material suplementario 3), no hubo diferencias sustanciales en comparación con lo hallado en los análisis principales. De igual forma, al evaluar los datos incluyendo sólo a las muertes que previamente se encontraban como casos, tanto a nivel global como por olas epidémicas (material suplementario 4), no se evidenció cambios en relación a la asociación principal reportada. Se evidenció que la diferencia fue significativa en todo momento al comparar el riesgo de muerte con la altitud de 0-500 msnm, siendo la categoría de 2001-3500 msnm la que consistentemente presentó un menor riesgo de muerte. Asimismo, los resultados principales también fueron consistentes al repetir las regresiones usando la clasificación de altitud alternativa, más alineada con los pisos altitudinales propuestos por Pulgar Vidal (material suplementario 5). Todo esto puede ser evidenciado en el material suplementario.

## DISCUSIÓN

Se encontró un menor riesgo de muerte por la COVID-19 en las categorías de altitud intermedia y moderadamente alta, al comparar contra la categoría de 0-500 msnm, aunque en la evaluación por olas epidémicas sólo se mantuvo asociada consistentemente la categoría de altitud moderadamente alta, no evidenciándose este menor riesgo de muerte por encima de 3500 msnm.

La relación entre altitud y mortalidad por COVID-19 ha generado resultados inconsistentes desde el inicio de la pandemia. Estudios iniciales en Latinoamérica sugirieron un efecto protector de la residencia a gran altitud sobre la mortalidad y el contagio ^10^^-^^12^, atribuyendo el efecto a la hipoxia hipobárica crónica ^27^^-^^29^. Sin embargo, estos estudios no siempre ajustaron por covariables relevantes ni consideraron la asincronía geográfica de la propagación del virus, especialmente considerando que los epicentros iniciales generalmente ocurrieron en zonas de baja altitud.

Estudios posteriores en Perú, utilizando datos provinciales y diseños ecológicos, no encontraron una tendencia protectora monótona. Se destacó que diferencias en densidad poblacional y otros factores sociodemográficos podrían explicar parcialmente los hallazgos ^16^^,^^20^. Estudios internacionales mostraron resultados mixtos; por ejemplo, en Estados Unidos, la letalidad disminuyó con la altitud tras ajustar por comorbilidades y variables sociodemográficas ^18^, mientras que en México los hombres sobre los 2000 msnm y las mujeres sobre los 2500 msnm presentaron mayor riesgo de muerte que habitantes por debajo de los 500 msnm ^17^. Esta heterogeneidad refleja la complejidad de los factores individuales y contextuales, y sugiere que la relación entre altitud y mortalidad podría no ser lineal.

Investigaciones recientes sugieren que la relación entre altitud y mortalidad podría seguir un patrón no lineal en forma de “U” ^30^, donde altitudes moderadas favorecen adaptaciones protectoras frente a la hipoxia, mientras que altitudes extremas podrían inducir cambios fisiopatológicos crónicos que aumenten el riesgo de mortalidad, como hipertensión pulmonar y alteraciones cardiovasculares ^(^^24^^,^^31^^,^^32^^).^ Nuestro estudio encontró evidencia de un efecto protector a altitudes intermedias, pero no en altitudes extremadamente elevadas, consistente con la hipótesis de una relación no monotónica. Este patrón se mantuvo incluso al emplear clasificaciones altitudinales basadas en los pisos ecológicos de Pulgar Vidal, sugiriendo que la tendencia observada no depende exclusivamente de los puntos de corte fisiológicos utilizados.

La plausibilidad fisiológica de estos hallazgos se apoya en mecanismos de adaptación a hipoxia crónica. Se ha planteado que la hipoxia crónica puede influir en la regulación de la enzima convertidora de angiotensina - 2 (ECA-2), el cual es el receptor celular de ingreso utilizado por el SARS-CoV-2 ^33^, y en la fisiología cardiovascular y respiratoria ^34^. No obstante, esta evidencia es preliminar y los mecanismos precisos que relacionan altitud, hipoxia y desenlaces por COVID-19 requieren estudios experimentales y clínicos específicos.

Si bien el mecanismo exacto del efecto protector de la altitud no puede determinarse con certeza, se sabe que la hipoxia hipobárica crónica induce adaptaciones cardiovasculares y respiratorias beneficiosas. En contraste, la administración aguda de eritropoyetina (EPO) en pacientes con COVID-19 no demostró eficacia ^(^^35^, probablemente por tratarse de intervenciones no adaptativas ni sostenidas. Teóricamente, intervenciones controladas que simulen hipoxia de manera segura, como el entrenamiento en altitud o la hipoxia inducida por ejercicio supervisado, podrían reproducir algunas ventajas adaptativas y beneficiar la salud cardiovascular y respiratoria en personas de diversas altitudes ^36^. No obstante, esta idea es especulativa y requiere estudios experimentales para evaluar su seguridad y eficacia.

Es importante mencionar que evitar el contagio y seguir las medidas de bioseguridad son y serán la mejor forma de garantizar la propia integridad ^8^. Nuestros hallazgos deben interpretarse considerando las limitaciones inherentes a los datos disponibles, incluyendo la ausencia de información sobre comorbilidades individuales, la heterogeneidad en la disponibilidad de recursos sanitarios y la imposibilidad de considerar cobertura de vacunación distrital. Estas limitaciones podrían introducir confusión residual y afectar la validez y confiabilidad de los resultados.

Debido a que existe un número indeterminado de casos asintomáticos, no es posible estimar con exactitud el riesgo de muerte sobre el total de infecciones, limitación compartida por todos los estudios basados en registros nacionales. Además, el uso de bases secundarias pudo conllevar sesgos por mala clasificación o registro incompleto de casos y defunciones, especialmente en distritos remotos con menor acceso a recursos diagnósticos. Adicionalmente, durante las primeras olas epidémicas, la capacidad diagnóstica y de vigilancia epidemiológica evolucionó rápidamente y de manera desigual en el territorio, lo que podría haber generado una detección diferencial de casos y muertes según el momento y la ubicación geográfica. Si la subnotificación difirió por altitud, tanto en casos confirmados como en defunciones, los estimados de letalidad podrían haberse visto afectados. Aunque la base oficial del MINSA incluye muertes que no habían sido registradas previamente como casos, persiste la posibilidad de captura diferencial por altitud. Por ello, los resultados deben interpretarse con cautela.

Los datos de disponibilidad de recursos de salud presentaron inconsistencias importantes y extensos periodos de tiempo sin reporte. Además, dado que la disponibilidad de camas de hospitalización, UCI, UCIN y camillas de emergencia es una variable altamente dinámica, se optó por exigir correspondencia específica entre el distrito y la fecha de cada caso para su incorporación analítica. Esta decisión metodológica, orientada a reducir el sesgo de medición temporal, incrementó la proporción de datos ausentes y limitó su uso en el análisis principal, de modo que sólo el 42,7% de los registros contaba con información válida. En consecuencia, estas variables no fueron incluidas en el modelo multivariable principal y se evaluaron únicamente en análisis de sensibilidad, los cuales se presentan en el material suplementario.

Asimismo, no fue posible incorporar comorbilidades individuales relevantes (como diabetes, hipertensión o cáncer), debido a la naturaleza del estudio y al uso de bases de datos públicas anonimizadas que no contienen información clínica detallada. Esta limitación constituye una fuente importante de confusión residual, dado que dichas comorbilidades son fuertes predictores de mortalidad por COVID-19 y podrían estar diferencialmente distribuidas según la altitud. De igual forma, no se contó con información sistemática y comparable sobre la cobertura de vacunación poblacional a nivel distrital y por periodo epidémico, lo que impidió evaluar posibles efectos indirectos de protección comunitaria (efecto de rebaño). La ausencia de esta variable podría introducir confusión contextual adicional, en la medida en que la intensidad de las estrategias de vacunación masiva pudo haber variado entre distritos. Finalmente, otros factores no medidos, como la contaminación ambiental o diferencias no capturadas en la infraestructura sanitaria, podrían estar influyendo en la asociación observada. No obstante, se incluyeron en los modelos todas las variables individuales y contextuales disponibles y relevantes para esta asociación, con el objetivo de minimizar la confusión residual.

Una limitación que se debe tener en consideración en relación con el análisis estratificado por ola epidémica es el uso de fechas de corte nacionales para definir las fases de la pandemia. La propagación del SARS-CoV-2 en Perú fue marcadamente asincrónica, con diferencias temporales importantes entre regiones de la Costa, la Sierra y la Selva. En este contexto, la estratificación por olas nacionales puede introducir confusión temporal, al comparar distritos que se encontraban en fases epidemiológicas locales distintos (inicio, pico o descenso) dentro de una misma “ola epidémica” definida a nivel país. En consecuencia, parte de las diferencias observadas en la letalidad podría reflejar variaciones en la intensidad de transmisión o en la presión sobre el sistema de salud, más que un efecto intrínseco de la altitud.

Se reconoce que la estructura de los datos presenta una jerarquía natural en la cual los individuos se encuentran anidados dentro de los distritos de residencia, lo cual introduce una correlación entre observaciones dentro del mismo cluster (distrito), por lo que se ajustó el análisis empleando una varianza robusta agrupada por distrito, con el fin de corregir explícitamente la dependencia intra-distrito al ajustar la matriz de varianza-covarianza teniendo en cuenta la estructura de clustering propia del diseño de los datos. No obstante, se resalta que este enfoque corrige la varianza de manera adecuada para la inferencia, pero no modela la variabilidad no observada entre distritos, como sí lo haría un modelo jerárquico o de efectos mixtos, lo que podría afectar la precisión de los intervalos de confianza y dejar una confusión contextual residual no capturada por factores relacionados con la altitud (como la calidad del sistema de salud, accesibilidad territorial, inversión pública, cohesión social, entre otros).

El uso de la altitud distrital como proxy de exposición fisiológica a la hipoxia hipobárica crónica implica inherentemente una simplificación. Aunque se categorizaron cuatro rangos basados en umbrales fisiológicos descritos en la literatura, esta aproximación podría introducir sesgo de mala clasificación de la exposición. En particular, la categoría ≥3500 msnm agrupa zonas ecológicas distintas (Suni y Puna) cuyos niveles de adaptación y respuesta fisiológica a la hipoxia pueden diferir. La unificación de ambos estratos pudo atenuar diferencias reales entre ellos y sesgar los estimados hacia la nulidad. Este aspecto se evaluó mediante un análisis de sensibilidad alternativo utilizando la clasificación altitudinal de Pulgar Vidal. Asimismo, la categoría 0-500 msnm incluye tanto áreas costeras con alta disponibilidad de servicios como zonas de la Selva Baja con menor oferta sanitaria. Esta heterogeneidad estructural podría generar confusión residual no completamente controlable, lo que se reconoce como otra limitación relevante del estudio.

Otra posible fuente de sesgo proviene de la movilidad de personas que requerían atención en centros ubicados a diferente altitud. Se decidió emplear la altitud del lugar de residencia en lugar de la del lugar de fallecimiento, dado que el efecto protector asociado a la hipoxia hipobárica crónica solo se produce con exposiciones sostenidas y prolongadas. Por ello, los desplazamientos para recibir atención no generarían un efecto fisiológico protector, e incluso un traslado agudo hacia zonas de mayor altitud podría representar un factor de riesgo más que un beneficio. Aun así, no puede descartarse completamente un error de medición derivado de este patrón de movilidad.

Dentro de las fortalezas se puede resaltar que es el estudio que abarca el mayor periodo de tiempo de análisis en Perú. Además, aunque la altitud puede variar dentro de un mismo distrito, en la mayoría de casos estas diferencias no superan los 1000 metros, magnitud pequeña en comparación con los más de 4000 metros observables entre distritos de una misma provincia, por lo que el potencial sesgo dentro de distrito sería menor en relación con el gradiente altitudinal nacional.

En conclusión, en Perú, durante el periodo 2020-2022, los datos no respaldan la hipótesis de una disminución monotónica del riesgo de muerte por COVID-19 con el incremento de la altitud de residencia. Por el contrario, el riesgo de mostró consistentemente un patrón no lineal en forma de “U”, con menor letalidad entre los casos residentes a altitudes intermedias (2001-3500 msnm) en todos los análisis realizados. Estos hallazgos sugieren que el papel de la altitud en la letalidad por COVID-19 es complejo y probablemente condicionado por procesos fisiológicos y contextuales, y refuerzan la necesidad de interpretar los factores geográficos en conjunto con otras determinantes estructurales y con la implementación sostenida de medidas de prevención y control en futuras epidemias.

Se recomienda que futuras investigaciones continúen explorando la posible relación no lineal entre la altitud de residencia y la muerte por COVID-19, incorporando enfoques analíticos que permitan modelar adecuadamente dicha no linealidad. Asimismo, estudios con diseños individuales y prospectivos deberían evaluar marcadores inflamatorios, hematológicos y otras proteínas relacionadas con la respuesta a la hipoxia, con el fin de contribuir a la comprensión de los posibles mecanismos biológicos subyacentes a las diferencias observadas según condiciones geográficas.
